# An upper bound on one-to-one exposure to infectious human respiratory particles

**DOI:** 10.1073/pnas.2110117118

**Published:** 2021-12-02

**Authors:** Gholamhossein Bagheri, Birte Thiede, Bardia Hejazi, Oliver Schlenczek, Eberhard Bodenschatz

**Affiliations:** ^a^Laboratory for Fluid Physics, Pattern Formation and Biocomplexity, Max Planck Institute for Dynamics and Self-Organization, Göttingen 37077, Germany;; ^b^Institute for Dynamics of Complex Systems, University of Göttingen, Göttingen 37077, Germany;; ^c^Laboratory of Atomic and Solid State Physics, Cornell University, Ithaca, NY 14853;; ^d^Sibley School of Mechanical and Aerospace Engineering, Cornell University, Ithaca, NY 14853

**Keywords:** SARS-CoV-2, COVID-19, infection risk, face mask, near-field model

## Abstract

There is ample evidence that masking and social distancing are effective in reducing severe acute respiratory syndrome coronavirus 2 (SARS-CoV-2) transmission. However, due to the complexity of airborne disease transmission, it is difficult to quantify their effectiveness, especially in the case of one-to-one exposure. Here, we introduce the concept of an upper bound for one-to-one exposure to infectious human respiratory particles and apply it to SARS-CoV-2. To calculate exposure and infection risk, we use a comprehensive database on respiratory particle size distribution; exhalation flow physics; leakage from face masks of various types and fits measured on human subjects; consideration of ambient particle shrinkage due to evaporation; and rehydration, inhalability, and deposition in the susceptible airways. We find, for a typical SARS-CoV-2 viral load and infectious dose, that social distancing alone, even at 3.0 m between two speaking individuals, leads to an upper bound of 90% for risk of infection after a few minutes. If only the susceptible wears a face mask with infectious speaking at a distance of 1.5 m, the upper bound drops very significantly; that is, with a surgical mask, the upper bound reaches 90% after 30 min, and, with an FFP2 mask, it remains at about 20% even after 1 h. When both wear a surgical mask, while the infectious is speaking, the very conservative upper bound remains below 30% after 1 h, but, when both wear a well-fitting FFP2 mask, it is 0.4%. We conclude that wearing appropriate masks in the community provides excellent protection for others and oneself, and makes social distancing less important.

## Significance

Wearing face masks and maintaining social distance are familiar to many people around the world during the ongoing SARS-CoV-2 pandemic. Evidence suggests that these are effective ways to reduce the risk of SARS-CoV-2 infection. However, it is not clear how exactly the risk of infection is affected by wearing a mask during close personal encounters or by social distancing without a mask. Our results show that face masks significantly reduce the risk of SARS-CoV-2 infection compared to social distancing. We find a very low risk of infection when everyone wears a face mask, even if it doesn’t fit perfectly on the face.

Infectious airborne diseases such as severe acute respiratory syndrome (SARS) 2002, measles, seasonal influenza, tuberculosis, and, more recently, coronavirus disease 2019 (COVID-19) caused by SARS coronavirus 2 (SARS-CoV-2) are transmitted via direct and indirect exposure from an infectious to a susceptible (1–4). One indirect route of transmission is via airborne transport of particles released from an infectious respiratory tract, that is, nasal/oral cavities, pharynx, larynx, trachea, and lungs–here we use the term particle(s) to refer to <1-mm particulate matter suspended in air, regardless of composition.

Human respiratory particles greatly vary in composition and size, and span several decades in length scale (e.g., see refs. 4 and 5, and references therein). Concentrations of exhaled particles and their size have been found to strongly depend on the type of respiratory activities, for example, speaking, or singing as compared to breathing. Vocalization-related respiratory activities, that is, sound pressure, peak airflow frequency, and articulated consonants, strongly influence particle emission (4, 5). Infectious respiratory particles may contain single or multiple copies of pathogens when exhaled by the infectious, and, when inhaled by the susceptible, there is a risk of infection given the absorbed infectious dose (3). Furthermore, relative humidity (RH) and temperature influence the drying and settling of wet particles by gravity when exhaled into the ambient (see refs. 3–5, and references therein). There is an ongoing debate about whether COVID-19 is transmitted primarily via aerosols or droplets (6, 7). There is also a longstanding debate about what is actually meant by aerosols or droplets (8). At their core, these debates are fueled by our inadequate understanding of how airborne disease transmission works, or, simply put, how exactly particles produced in the respiratory tract of the infectious become airborne, how they change in the ambient, and where and in what quantity they deposit in the respiratory tract of the susceptible. As simple as it may appear, the detailed mechanisms involved in each part of these processes are extremely complex (4).

On the source side, that is, the infectious, we have the physioanatomical dependency intertwined with the complexity of particle production controlled by the respiratory maneuver, and the size-dependent pathogen concentrations due to differences in their origin sites and/or their volume and/or the nature of the pathogen itself. In the ambient air, we have the turbulent cloud of particles exhaled by the infectious while being turbulently diffused and advected into the ambient. The advection and turbulent diffusion of the exhale cloud is strongly influenced by the ambient thermodynamical conditions and airflow, for example, the type and strength of the ventilation in indoor environments, or wind conditions outdoors as well as other natural air currents that carry away the exhale. While being advected, respiratory particles may lose their volatile content due to evaporation, which is determined by their chemical composition, ambient thermodynamical conditions, exhale flow velocity, mixing with the ambient air, and the time they need to reach an equilibrium size (4, 9). While being advected with the flow, some particles will be lost due to deposition on nearby surfaces depending on their size, shape, density, and charge and might get resuspended at a later time. In addition, pathogens might lose their infectiousness before being absorbed by the susceptible. On the receptor side, that is, the susceptible, the mechanisms controlling the inhalability and absorption of pathogen-laden particles depend not only on the physical–anatomical properties of the receptor but also on the receptor’s breathing maneuver, inhaled particle size and composition, their rehydration growth rate due to condensation in the receptor’s airways, and the velocity, temperature, and RH of the inhaled air.

It can be concluded that airborne disease transmission is a problem that involves complex physical processes across a wide range of spatial and temporal scales, making it very difficult to predict its subsequent course with an acceptable degree of certainty. Uncertainties about airborne transmission routes and mechanisms arguably were the main reason for the differences in infection control measures implemented in different countries. A notable example is the use of face masks, which were initially discouraged to the public or recommended only for symptomatic cases and health care workers in the United States (10). Another example is the debate about the effectiveness of social distancing in reducing the risk of transmission (see, e.g., refs. 11 and 12). This is despite the fact that masking and/or social distancing in the community have been shown to be very effective in reducing the risk of SARS-CoV-2 infection in many East Asian countries (see e.g., refs. 13–16). There is also ample evidence from laboratory experiments (17), and metaanalyses and literature syntheses (11, 18–20) showing that masking and/or social distancing are effective in reducing SARS-CoV-2 transmission.

During the COVID-19 pandemic, significant progress was possible on the medium problem, considering the source–medium–receptor trilogy, by greatly simplifying the dispersion of infectious aerosols using the well-mixed room assumption (e.g., refs. 3, 12, and 21–25). These models assume the pollutants (i.e., respiratory particles) to be instantaneously diluted and completely mixed throughout the room volume before they reach the receptor. As a result, the concentration of particles is the same at all locations in the room and decreases exponentially with time, which depends on the room air exchange rate, deposition rate, filtration rate, and particle sizes. With this assumption, it is possible to study the mean properties in a room without having to consider turbulent fluctuations, transport by flow, or other location dependencies to estimate the far-field infection risk. In practice, however, there will be concentration fluctuations in a room even if the airflow in the room is a fully developed turbulence. The well-mixed room assumption cannot predict the risk of infection when the room volume is large or the distance between infectious and susceptible is small. In such cases, exposure in the near field (i.e., close range) must be taken into account, where the pathogen concentration will be much higher than that predicted by the well-mixed room model.

There are many everyday encounters where individuals are exposed in the near field, whether it is a brief interaction with the cashier at the supermarket, lunch with a colleague, waiting in a line, talking, singing together, exercising, or others. In these encounters, both individuals or one of them may wear face masks, they may use masks of different types or fits, or they may simply maintain social distancing.

While much has been learned in this context from insightful studies (3, 4, 11, 17–19, 21, 22, 26–28), our work goes beyond and introduces the upper bound on exposure/infection risk as a quantifier that can guide infection control measures. While our quantitative analysis is limited to typical parameters for SARS-CoV-2, our approach also applies to other parameters and other respiratory infectious diseases. Here we answer the following questions:

• What is the upper bound on SARS-CoV-2 infection risk for near-field exposure?
• How does this upper bound change with the respiratory activities, that is, passive breathing vs. talking?
• How does this upper bound vary with the exposure duration?
• How do the type of face mask and the way it fits to the face affect the upper bound?
• Which intervention strategy, between masking and social distancing, is most effective?

## The Upper Bound on Exposure/Infection Risk

The infection risk is a function of the absorbed pathogen dose *μ*, which is fully defined in Eq. **3** and can be regarded as the “effective exposure,” but can be simplified here to introduce the concept of the upper bound,

[1] $$\mu \propto n_{I}\times \mathrm{TOL}\times f_{d}\times \mathrm{TIL}\times D_{rt},$$

where *n_I_* is the pathogen number concentration produced by the infectious, *TOL* is the total outward leakage of the face mask worn by the infectious, $1-f_{d}$ represents the reduction in concentration of infectious particles due to dilution and deposition in the environment between infectious and susceptible, *TIL* is the total inward leakage of the face mask worn by the susceptible, and *D_rt_* is the intake/deposition efficiency of the susceptible respiratory tract. For infectious (or susceptible) individuals without a face mask, *TOL* (or *TIL*) is equal to 1.0. For the infectious with a face mask, *TOL* is a function of particle diameter at the time of exhalation, *d*_0_. For the susceptible with a face mask, *TIL* is a function of the particle diameter at the time of inhalation, *d_e_*, which will be, in most situations, smaller than *d*_0_, as a result of evaporation in the ambient. At an RH of 30%, which is rather low for typical indoor spaces, *d_e_* should stabilize at a quarter of *d*_0_; that is, shrinkage factor $w=d_{0}/d_{e}=4$ (see figure 2B in ref. 5).

The fraction ratio *f_d_* is the most difficult parameter to determine, as it must take into account the combined effect of dilution of the exhaled air with the ambient, deposition losses, and pathogen inactivation. It depends on the size of exhaled particles, respiratory activity, shrinkage factor due to evaporation, advection distance/time from the infectious to the susceptible person, room conditions (RH, temperature, airflow, type of ventilation), anatomical and physiological characteristics of the infectious/susceptible persons, and whether or not the infectious person is wearing a face mask (as this significantly affects exhalation flow), and the biological properties of the pathogen. Therefore, it is very difficult, if not impossible, to make a detailed prediction on the situational risk of infection during a one-to-one exposure. Even if one knew an example, the situational variability is so large that exemplary knowledge can hardly be generalized. Thus detailed examples may not help much in guiding infection control measures.

**Fig. 1.**
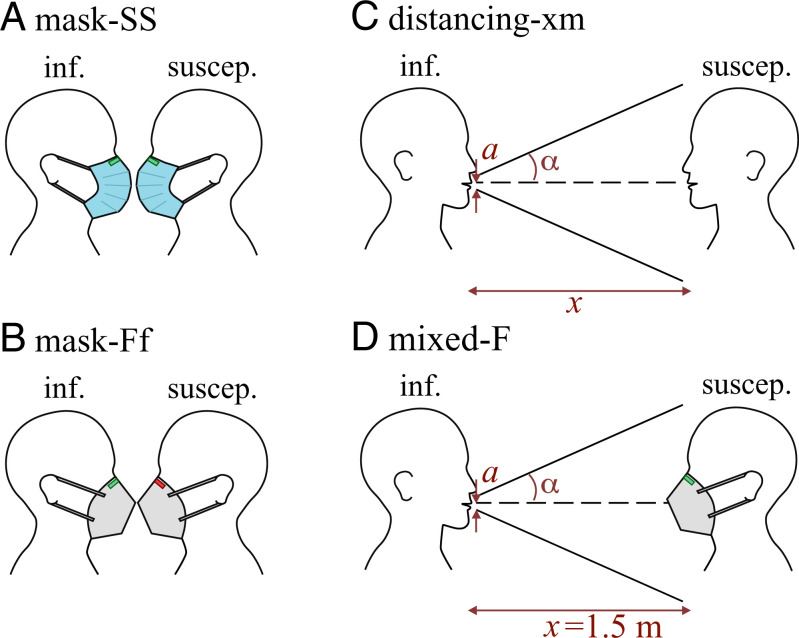
Schematics of scenarios investigated in this study. (*A* and *B*) The mask-is scenario: a masked infectious breathing/speaking to a breathing-only masked susceptible, where the susceptible is exposed to the nondiluted total outward leakage of the infectious exhale; i and s indicate the type of mask worn by the infectious and susceptible individuals, respectively, with adjusted (i.e., well-fitted to the face) FFP2 mask abbreviated by “F,” FFP2 mask without adjustment (i.e., without fitting to the face) abbreviated by “f,” and adjusted surgical mask abbreviated by “S” (only mask-Ff and mask-SS are sketched here). For this scenario, $f_{d}=1.0$. (*C*) The distancing-xm scenario: an unmasked breathing-only susceptible exposed to the exhalation cone of an unmasked breathing/speaking infectious while the distance between the two is *x* meters. For this case, *f_d_* is calculated via the exhalation cone formula $f_{d}=a/(x\;\tan(\alpha ))$, where a= 1.8 cm is the radius of the mouth and α= 10^∘^ is the exhalation cone half-angle. (*D*) The mixed-s scenario: the same as *C*, but susceptible is wearing a mask and the distance is kept fixed at 1.5 m; *s* indicates the type of mask worn by the susceptible. Cases considered for this scenario are “mixed-S” and “mixed-F,” which correspond to susceptible wearing adjusted surgical and adjusted surgical FFP2 mask, respectively (only mixed-F is sketched here). For this scenario, *f_d_* is calculated based on the exhalation cone formula similar to the distancing scenario. Different types of masks and fittings are shown in Fig. 2 and will be discussed later.

Progress can be made by defining situations for which the exposure/infection risk is an upper bound. The main idea behind the upper bound is that, if a scenario proves to be safe under the upper bounds defined here, there is no question of its effectiveness under real conditions. Here we present three scenarios (Fig. 1): 1) the mask scenario, in which everyone wears a face mask and the susceptible exposed to the concentration of particles that could penetrate through the infectious mask, e.g., mask-FF or mask-SS if both are wearing an adjusted FFP2 mask or surgical mask, respectively, and mask-FS if the infectious is wearing an adjusted FFP2 mask and the susceptible is wearing a surgical mask; 2) the distancing scenario, in which no one wears a face mask and the susceptible inhales the turbulent-diluted exhalation of the infectious at some distance, e.g., distancing-1.5 m if they are located at a distance of 1.5 m from each other; and 3) the mixed scenario, which is similar to the distancing-1.5 m scenario, but the susceptible person wears a face mask; e.g., Mixed-F or Mixed-S scenarios when the susceptible is wearing an adjusted FFP2 or surgical mask, respectively.

Given these scenarios and our goal of calculating an upper bound on exposure, we can distinguish between the following two situations to calculate *f_d_*:

1. The infectious does not wear a face mask. Following Yang et al. (26), the susceptible can be assumed to be within the turbulent-diluted exhalation cone of the infectious. The susceptible then inhales this air without a face mask (the distancing scenario) or with a face mask (the mixed scenario) and absorbs some pathogen-borne particles. With the knowledge of disease-dependent viral load and infectious dose, the risk of infection for susceptible can then be calculated. This constitutes an upper bound on exposure/infection risk, as it assumes that the susceptible is stationary in the exhalation cone for the duration of the encounter, the ambient air is still and no other airflow exists, and there is no particle deposition and/or pathogen inactivation. Clearly, this will not be the case in most situations; however, this upper bound can serve as a much needed guidance. In this situation the exhalation cone formula $f_{d}=a/(x\;\tan(\alpha ))$ is used, where *x* is the distance between the source and the receptor, *a* is the radius of the mouth (assuming a circular shape), and *α* is the exhale jet’s half-angle. We can assume a= 1.8 cm and α= 10^∘^, which gives $f_{d}=0.1$ at a distance of 1 m as a conservative upper bound estimate; see *Materials and Methods* for more details.
2. The infectious and susceptible are both wearing a face mask (mask scenario). It is well known that face masks, for example, FFP2, KN95, and surgical masks, differ not only in their filter material penetration properties but, even more importantly, in the leakage from the face mask seal. In this situation, it is difficult, if not impossible, to define an exhalation cone, as the direction of leakage and exhalation through the mask itself depends on the spatiotemporal exhalation/inhalation dynamics of the specific mask design under the specific respiratory situation. Despite all this, a well-defined and useful upper bound can be calculated by setting $f_{d}=1.0$. In practice, *f_d_* is likely to be well below 1.0, even if the susceptible is standing directly next to the masked infectious, as part of the exhalation cone may be directed away from the susceptible, for example, most likely upward at the nose for FFP2 masks or sideways and upward, as is typical for surgical masks.

The *n_I_* is calculated based on the multimodal fits published by Bagheri et al. (5) without correcting for the infectious age, assuming SARS-CoV-2 viral load of 10^8.5^ mL^−1^ as discussed in *Infection Risk Model*. Bagheri et al. (5) determined their fits based on measurements on 132 healthy volunteers aged 5 y to 80 y during breathing and vocalization. They used multiple aerosol spectrometers and in-line holography to cover a particle size range of 50 nm to 1 mm. Here, particles of <50 μm are considered for the calculation of infection risk, unless otherwise stated, as we consider this to be a conservative and realistic limit based on recent numerical simulations (9, 29). Particles larger than 50 μm are unlikely to travel the distances of ≥1.5 m investigated in this study or to escape from the infectious face mask, as will be shown later. It should be noted that, from here on, for the particle size range considered, we do not take into account any losses, for example, due to settling by gravity or inactivation of the pathogens, as our aim is to calculate the upper bound on the risk of exposure/infection. In addition, for all scenarios considered here and regardless of the distance between the infectious and the susceptible, the shrinkage factor *w* is assumed to be four (5). This leads to a higher estimate of the infection risk than that with *w*  <  4.

We have measured the *TIL* in this study on human subjects, as the available data in literature are mostly based on measurements on manikins (30–40), and little or no information regarding the dependency of leakage on particle size is known for human subjects (41–48). The few studies with human subjects that present size-dependent data (49–52) are performed with different types of face masks, and none of these covers a representative particle size range necessary for risk calculations considered here. For the lack of a reliable measurement method on human subjects and since data from the literature are inconclusive, we assume the *TOL* to be the same as *TIL* (see *Total inward leakage* and *Total outward leakage* for more details).

*D_rt_* is calculated based on the model developed by The International Commission on Radiological Protection (ICRP) (53). For calculating *D_rt_*, we have also taken into account the time-dependent hygroscopic growth of particles in the respiratory tract of the susceptible. Further details of all the models, input parameters, and assumptions are presented under *Materials and Methods*.

The following sections first present the results of mask efficacy/leakage measurements, followed by discussions of the combined effect of mask leakage and respiratory tract deposition. Finally, the risk of SARS-CoV-2 infection under different masking, social distancing, or a combination of these scenarios is presented, and consequences are discussed.

### Face Mask Efficacy

Total Inward leakage is defined as $\mathrm{TIL}=q_{P,in}P_{in}+q_{L,in}L_{in}$, where *P_in_*  =  *P_filter_* is the inward penetration through the mask fabric and *L_in_* is the penetration through face seal leakage. The $q_{P,in}$ and $q_{L,in}$ are the ratio of the flow rate through the mask fabric and face seal leaks, respectively, to the total flow rate into the mask. As already mentioned above, we have measured filter penetration *P_filter_* and total inward leakage *TIL* exemplary on human subjects. The results for these measurements are presented below.

#### Filter penetration

Excellent data on filter penetration can be found in the literature; see, for example, refs. 47, 51, and 54–61. We performed our own measurements of filter penetration using the same instruments and diffusion dryers that we used in our *TIL* measurements. For filter penetration measurements, we have also used, additionally, a TSI NanoScan Scanning Mobility Particle Spectrometer 3910 (SMPS) to measure sizes smaller than 300 nm. More details for the setup are presented in *SI Appendix*, section 1.C. The experiments were performed for the same particle size ranges (bins) for the TSI Optical Particle Sizer Model 3330 (OPS) from 300 nm to 10 μm while using the same dolomite dust as test particles. Our results from the OPS instruments (and SMPS) agree well with the penetration values reported in the literature, indicating that our experimental procedures used for the *TIL* measurements are sound. For the sake of completeness, we report the main results here. We measured the filter penetration of three cloth, eight surgical, and five FFP2 masks. The examined cloth masks have the highest filter penetration, with a maximum of, on average, 85% for 0.3-μm particles. The surgical masks performed better, that is, showed lower filter penetration throughout but seem to fall into two categories. Four of the examined masks are close to the cloth masks, whereas the other four have filter penetrations of <12% throughout. The filter penetrations of the examined FFP2 masks are all below the 6% limit set by the EN 149:2001+A1:2009 standard (62). Both the surgical and the FFP2 mask used in the leakage experiments have filter penetrations below the mean we found for each category. Detailed results and comparisons with the literature are presented in *SI Appendix*, section 2.A.

#### Total inward (and outward) leakage

**Fig. 2.**
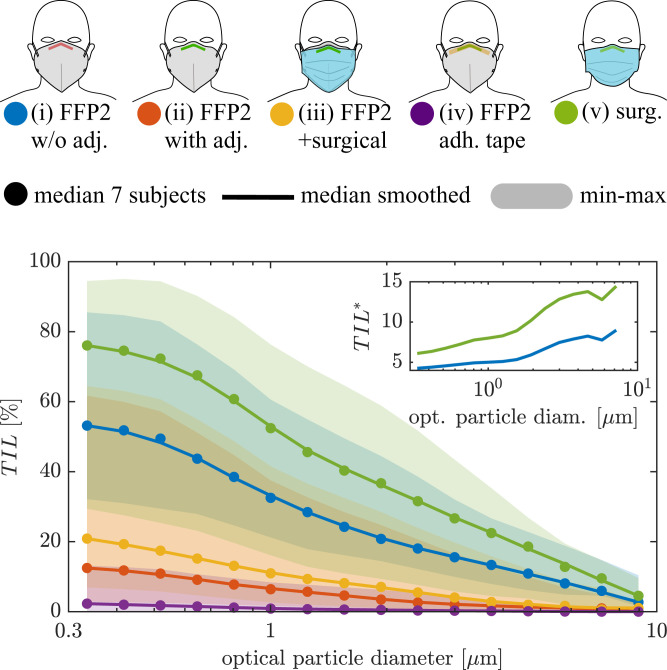
Median of the total inward leakage over all subjects for different mask-wearing cases. Smoothed curves are the three-point moving average. Shaded areas show minimum and maximum as an indication of variability in total inward leakage for different subjects–the individually measured particle size–dependent *TIL* can be found in *SI Appendix*, section 2.I. The first–last bin total leakage values are (*i*) 53.2 to 2.7%, (*ii*) 12.5 to 0%, (*iii*) 20.9 to 1.0%, (*iv*) 2.3 to 0%, and (*v*) 76.0 to 4.5%. *Inset* shows the total inward leakage of the surgical mask and the FFP2 mask without adjustment normalized with the total inward leakage of the adjusted FFP2 mask $TIL^{*}=\mathrm{TIL}/TIL_{\text{FFP2},\text{adj}.}$.

The median total inward leakage of seven subjects for mask cases *i* to *v* is shown in Fig. 2. Details of the measurement setup, mask dimensions, and fitting procedure are presented in *Total inward leakage*. The shaded regions represent the range of leakage values from the worst-performing to the best-performing mask/subject combination. The total inward leakage decreases for all mask wearing cases *i* to *v* with particle size for particles larger than 300 nm, which agrees well with the literature (30, 32, 33, 35, 49–51). The best mask fit, that is, least leakage, is found in case *iv*, in which the face seal leakage at the nose is eliminated by using double-sided adhesive tape 3M-1509 as explained in *Total inward leakage*. This indicates the strong influence of leakage at the side of the nose, which agrees with infrared observations on N95 masks (63), results from simulation studies (63, 64), and observations of tracer particles with half-mask respirators by Oestenstad et al. (65). In another investigation by Oestenstad and Bartolucci (66), however, leaks at the cheeks were found to play a significant role as well, which can possibly be explained by considering the fact that facial dimensions play an important role in leak positions (63, 66). Overall, our findings agree with the literature on total inward leakage, but we find higher leakages for an adjusted mask than most other studies investigating mask leakage on human subjects. More detailed comparisons with existing leakage measurements on human subjects can be found in *SI Appendix*, section 2.F.

Due to the high filter efficiency of the FFP2 mask (i.e., as shown in *SI Appendix*, Fig. S5), the majority of the particle concentration penetrating into the mask is caused by the face seal leakage. Wearing an FFP2 mask without any adjustment whatsoever, case *i*, leads to total inward leakage of 53% for the smallest particle bin (0.3 μm to 0.37 μm), which decreases to 16% for 3 μm. By simply adjusting the mask nosepiece to the nose, case *ii*, the mask’s *TIL* is improved by a factor of 4.3 for the smallest particles and by a factor of 7.5 for 3 μm particles (Fig. 2, *Inset*).

The surgical mask, however, is associated with the highest total inward leakage, with the maximum value being in excess of 70% occurring for the smallest particle size. This is caused both through relatively high filter penetration (5% for particles around 0.3 μm as shown in *SI Appendix*, Fig. S5) and the evidently high leakage. The total inward leakage of the surgical mask shows a similar trend to that of the FFP2 mask without adjustment and is 6 times higher compared to the adjusted FFP2 mask for the smallest particles and over 12 times higher for particles of >3 μm (Fig. 2, *Inset*). Thus, a better-fitting mask has a higher relative protection from large particles compared to small particles.

Wearing an additional surgical mask on top of the adjusted FFP2 masks, case *iii*, seems to have an overall negative effect on the total inward leakage compared to case *ii*. The effect of double filtering is negligible, as the filtration efficacy of the FFP2 mask is already large ( >99.98% for particles of >0.3 μm). A possible explanation for the decreased protection (increased inward leakage) could be that the additional pressure on the FFP2 mask caused by the surgical mask distorts the FFP2 mask and therefore causes new face seal leaks, or the increased breathing resistance leads to more leakage (67). For some individual subjects, however, the surgical mask on top of the FFP2 mask leads to a slight improvement, that is, decreased total inward leakage. In our early experiments, in which no diffusion dryer was used, we have observed an overall improvement for case *iii* over case *ii*. We therefore judge the results for wearing a surgical mask on top of an FFP2 mask as interesting, but, at this point, inconclusive. Facial hair is found to increase the face seal leakage and therefore total inward leakage slightly (*SI Appendix*, Fig. S11).

We found an increased *TIL* when subjects were mouth breathing compared to nose breathing for cases *i* and *ii*. With the mask taped at the nosepiece, case *iv*, we observed the exact opposite (cases *iii* and *v* were not investigated for mouth vs. nose breathing experiments; *SI Appendix*, Fig. S9). Thus the impact of nose vs. mouth breathing on the leakage remains inconclusive, and experiments on more subjects are necessary. Reading with a loud voice ( ∼ 80 dBA to 90 dBA) is found to decrease the *TIL* as compared to breathing through the nose by as much as a factor of 3 for the adjusted FFP2 mask (*SI Appendix*, Fig. S6). As a result, leakage values measured during breathing experiments are most likely an upper estimate for activities that are not associated with significant movement of the mask relative to the face.

For particles with diameter 0.1 μm to 0.3 μm, smaller than what we measured, the total inward leakage can be expected to plateau (cf., e.g., refs. 50 and 51). For larger particles than measured, >10 μm, the total inward leakage can be assumed to smoothly drop to zero–values of *TIL* for particles outside the OPS detection range are obtained by linear extrapolation, which are then smoothed by a linear Savitzky–Golay filter with a window of length five to maintain the signal trend. Zero-leakage median can already be observed for the largest measured particles with average diameter of 9 μm for the adjusted and taped FFP2 mask (cases *ii* and *iv*). Similar assumptions were used by Hinds and Bellin (68). As explained in *Materials and Methods*, the total leakage to the outside during exhalation is $\mathrm{TOL}=q_{P,ex}P_{ex}+q_{L,ex}L_{ex}$, where $q_{P,ex}$ and $q_{L,ex}$ are the flow ratios through filter and face seal leaks, respectively, during exhalation. In the absence of a suitable measurement procedure and given that available data in the literature are inconclusive on this topic (see *Total outward leakage*), we assume that the *TOL* is equal to the *TIL*.

### Effective Respiratory Tract Penetration

The overall effects of outward and inward mask penetration, as well as the transient particle size that depends on environmental conditions and time since exhalation, must be considered in order to capture the true particle penetration from infectious to susceptible. In the presence of particle shrinkage in the ambient, that is, *w* = 4, the penetration curves through susceptible mask fabric shown in *SI Appendix*, Fig. S5 in inhalation *P_in_* are shifted to the right compared to that of the infectious mask fabric in exhalation *P_ex_*. The same is true for face seal leakage during exhalation *L_ex_* and inhalation *L_in_*. As a result, the combined penetration in mask scenarios is rather complicated to estimate.

**Fig. 3.**
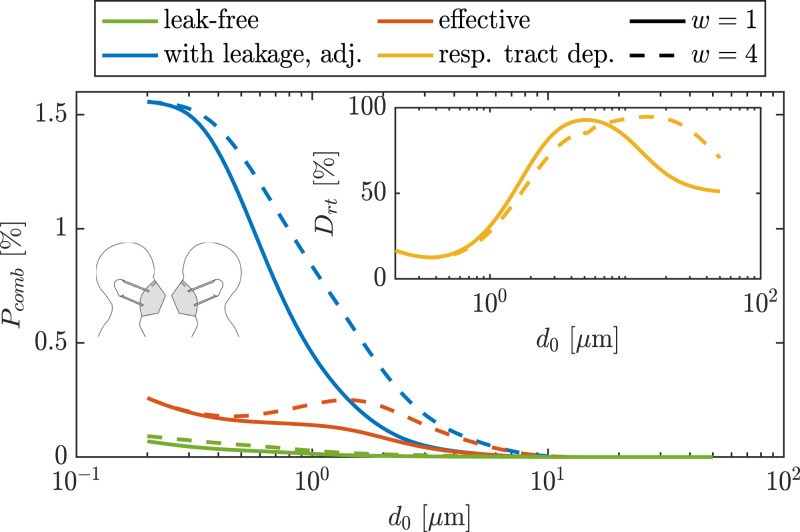
Combined penetration values when both infectious and susceptible are wearing FFP2 masks, that is, mask-FF scenario (combined penetration for mask-SS scenario is shown in *SI Appendix*, Fig. S14), and at different shrinkage factors of *w* = 1 (solid lines), that is, no shrinkage, and *w* = 4 (dashed lines) as a function of particle diameter at exhalation, that is, wet diameter *d*_0_. “Leak-free” curves correspond to $P_{ex}P_{in}$, “With leakage, adj.” curves correspond to TOL×TIL, and “effective” curves correspond to $\mathrm{TOL}\times \mathrm{TIL}\times D_{rt}$. Respiratory tract deposition *D_rt_* is shown in *Inset* for *w* = 1 and *w* = 4.

If we only consider ideal leak-free masks, that is, $q_{\mathrm{Pin}/ex}=1$ and $q_{\mathrm{Lin}/ex}=0$, the combined penetration through the fabrics of infectious and susceptible FFP2 masks $P_{in}P_{ex}$ is very low and is slightly affected by the shrinkage ratio, as shown in Fig. 3. In practice, however, masks are always associated with leakage. The combined penetration of adjusted FFP2 masks with leakage taken into account, that is, $\mathrm{TIL}\times \mathrm{TOL}=\left(q_{P,in}P_{in}+q_{L,in}L_{in}\right)\;\left(q_{P,ex}P_{ex}+q_{L,ex}L_{ex}\right)$, is much higher than that of leak-free masks for the whole range of particle sizes, as shown by the blue curves in Fig. 3. The impact of shrinkage factor is also more visible for the leak-included than leak-free penetration. The combined penetration for FFP2 masks with leakage increases with particle shrinkage factor, since large particles that penetrate through the mask of infectious have higher penetration probability through the mask of susceptible as they get smaller by a factor of *w*.

However, for investigating the exposure/infection risk, the combined penetration should also take into account the deposition in the respiratory tract of the susceptible *D_rt_*, which is shown in Fig. 3, *Inset*. The *D_rt_* for the case when *w* = 1 is based on the original model provided by the ICRP model (53). For *w* = 4, however, we have assumed particles undergo an unsteady hygroscopic growth as they enter respiratory tract of the susceptible, and, hence, the deposition fraction in different regions of the respiratory tract behaves differently, as shown by the dashed line in Fig. 3, *Inset*. Besides hygroscopic growth, particle shrinkage increases the inhalability probability of particles with initial wet diameter of >7 μm. In addition, particles with wet diameters of 1 μm to 3 μm would have about 10% lower probability of deposition when *w* = 4.

The combined effect of masks with leakage and the respiratory tract deposition, that is, the effective penetration $=\mathrm{TOL}\times \mathrm{TIL}\times D_{rt}$, is also shown in Fig. 3. It can be seen that, for *w* = 4, the maximum penetration occurs for ∼ 1.5-μm particles, whereas, in case of no shrinkage (i.e., *w* = 1), the maximum penetration occurs for the smallest particle size. This is an important difference, since the absorbed pathogen dose scales with the volume $d_{0}^{3}$ of the particles. For surgical masks, the results follow the same trends as shown in Fig. 3, but the penetration magnitudes are much higher (*SI Appendix*, Fig. S14).

### Exposure/Infection Risk for COVID-19

Details of the infection risk model, the relevant assumptions, and justifications for all the input parameters are presented in detail in *Infection Risk Model*. In the following discussions, we assume the FFP2 and surgical masks investigated in our study have characteristics comparable to any other FFP2 and surgical masks in terms of total inward and outward leakages and fitting on the subjects’ faces.

**Fig. 4.**
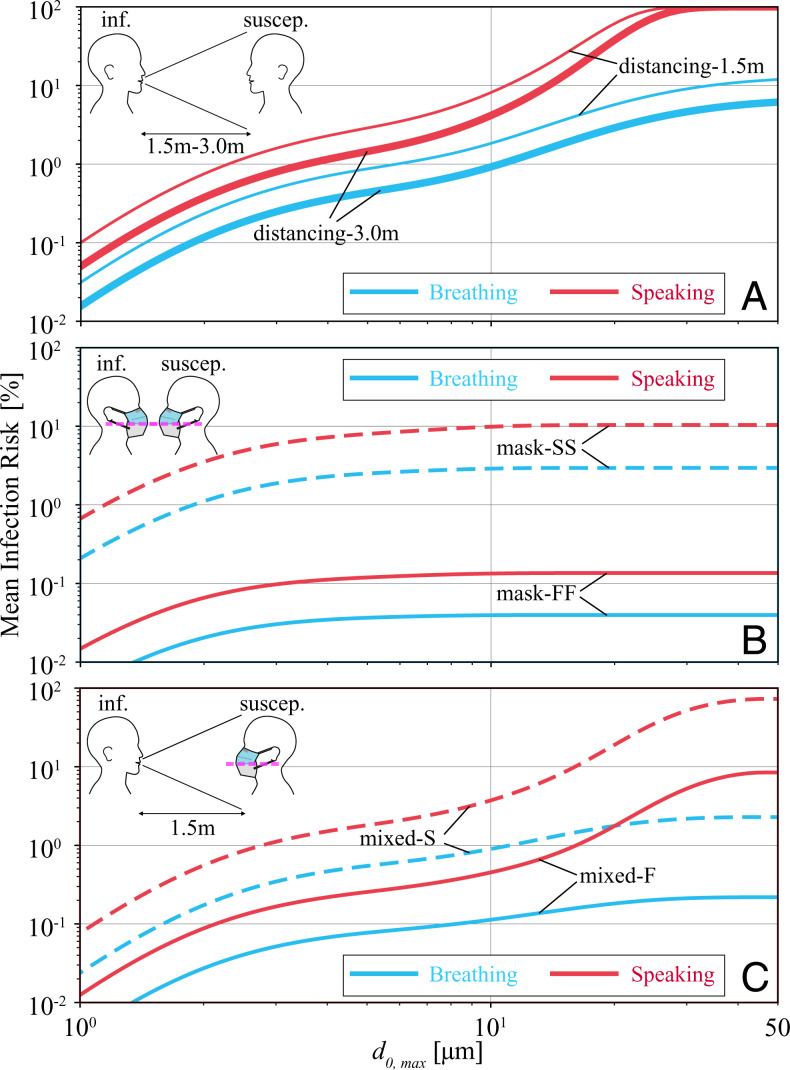
Mean risk of infection as a function of (wet) exhale diameter cutoff $d_{0,\max}$ when an infectious is breathing or speaking toward a breathing-only susceptible for a duration of 20 min considering (*A*) distancing, (*B*) mask, and (*C*) mixed scenarios. Other parameters used are *w* = 4, viral load $\rho_{p}=10^{8.5}\;$ virus copies per mL, and ID${}_{63.21}$ = 200. Details of scenario-specific parameters, for example, *f_d_*, are presented in the caption of Fig. 1.

Fig. 4 shows the mean risk of infection for the different scenarios and for a duration of 20 min as a function of wet diameter cutoff $d_{0,\max}$ (the diameter above which the particles are assumed to deposit before reaching the susceptible or get filtered by the infectious’ mask). The first observation that can be made is that, with a 5-μm cutoff, the typical cutoff size for aerosols (8), risk of infection is below 10% for all the scenarios. However, at cutoff size of 50 μm, which, with *w* = 4, translates to an equilibrium diameter $d_{e}=12.5\;$ μm, risk of infection increases significantly for distancing and mixed scenarios, particularly when infectious is speaking. While the trend of mean infection risk for breathing and speaking infectious is very similar for a cutoff size of 10 μm, significant deviations for larger cutoff sizes are visible in Fig. 4 *A* and *B*. The reason for such a significant deviation between breathing and speaking is the increased probability of producing >10 μm for vocalization-associated activities ([5]). However, this deviation in the trend disappears for masking scenario, Fig. 4*B*, in which the 10-μm cutoff is enforced by the masks themselves, as shown in Figs. 2 and 3. Fig. 4 *A* also shows that increasing distancing from 1.5 m to 3.0 m reduces risk of infection when infectious is breathing but not for a speaking infectious. In contrast, universal masking is an extremely effective strategy in reducing risk of infection, as shown in Fig. 4*B*, even for the absolutely extravagant scenario considered here, in which the susceptible is exposed to undiluted air penetrating through the fabric and leaks of the infectious’ face mask (i.e., $f_{d}=1.0$ in Eq. **1**). When both infectious and susceptible wear FFP2 masks, that is, Fig. 4*B*, a reduction in the risk of infection by a factor of ∼75 is expected compared with the case where both wear surgical masks. Other mask type/leakage combinations are discussed later. Finally, Fig. 4*C* shows that, when only the susceptible adheres to masking and even when they are distancing, the probability of infection risk can be as high as ∼10% if the susceptible wears an adjusted FFP2 mask, or ∼70% if the susceptible wears a surgical mask while in the exhalation cone of a speaking infectious. Fig. 4*C* shows that an adjusted FFP2 mask reduces risk of infection by about a factor of  10 compared to an adjusted surgical mask, independent of the infectious activity.

The distance traveled and residence time of even >50-μm particles in the air have been shown to be much larger than those usually considered (9, 29, 69). Thus, the mean infection risks shown in Fig. 4 at $d_{0,\max}=\;$ 50-μm cutoff are very plausible upper limits. Considering a larger particle cutoff would mainly affect the scenarios in which infections do not wear a face mask and speak, which are already unsafe even at $d_{0,\max}=\;$ 50-μm cutoff. Therefore, in the following, we restrict ourselves to the results obtained for this cutoff.

**Fig. 5.**
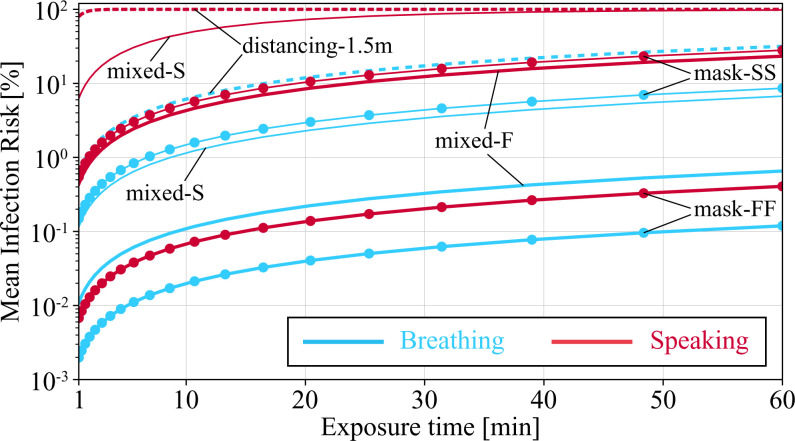
Mean risk of infection for a breathing-only susceptible to be exposed to a breathing or speaking infectious in different scenarios as a function of time and for diameter cutoff of 50 μm. Other parameters used for generating results shown in this plot are $d_{0,\max}=50\;\mu \text{m}$, *w* = 4 viral load, $\rho_{p}=10^{8.5}\;$ virus copies per mL, and ID${}_{63.21}$ = 200. Details of scenario-specific parameters, for example, *f_d_*, are presented in the caption of Fig. 1.

Fig. 5 shows risk of infection as a function of exposure duration for different scenarios. Let us consider an infection risk of 1% as the threshold beyond which a given scenario is unsafe. Given this threshold, the distancing scenarios quickly becomes unsafe, and, already after about 1.5 min for a speaking infectious, the risk of infection for the susceptible at a distance of 1.5 m is 90%. The next high-risk scenario is the mixed-S scenario with a speaking infectious, which surpasses the 1% threshold in less than a minute and reaches the 90% threshold after half an hour. All the speaking infectious scenarios with the exception of mask-FF bypass the 1% threshold within a few minutes and reach >10% in 1 h. The only breathing infectious scenario associated with >10% infection risk in 1 h is the distancing-1.5m scenario. The safest scenarios that stay below the 1% threshold for 1 h of exposure in order of best to worse are mask-FF for breathing and speaking infectious, respectively, followed by the mixed-F with a breathing infectious. Interestingly, the extremely conservative estimate of mask-FF with speaking infectious is safer than mixed-F with a breathing infectious. This is yet another indicator showing the effectiveness of universal masking, which is consistent with real-world observations (e.g., refs. 11, 13–16, 18, and 19).

**Fig. 6.**
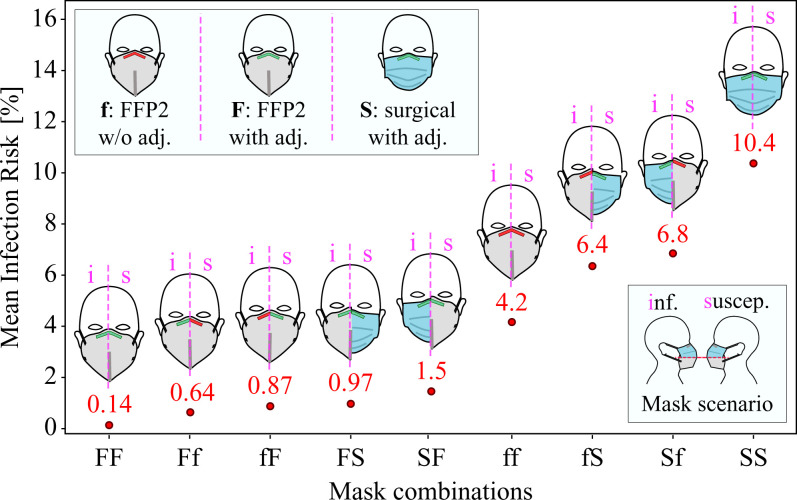
Mean risk of infection in mask scenarios with different mask combinations for a duration of 20 min. The horizontal axis shows the combination of masks used by the infectious and susceptible with two characters; the first character corresponds to the type of mask worn by the infectious, and the second character corresponds to that of susceptible. Mask types and fittings are abbreviated as follows: f, FFP2 mask without adjustment (Fig. 2, case *i*); F, FFP2 mask with adjustment (Fig. 2, case *ii*); S, surgical mask with adjustment (Fig. 2, case *v*). Other parameters used for generating results shown in this plot are $f_{d}=1.0,\;d_{0,\max}=50\;\mu \text{m}$, *w* = 4, viral load $\rho_{p}=10^{8.5}\;$ virus copies per mL, and ID${}_{63.21}$ = 200.

Fig. 6 shows different combinations of FFP2 fittings (F: with nosepiece adjustments and f: without nosepiece adjustments) and nosepiece-adjusted surgical masks (S) for a speaking infectious considering mask scenario and an exposure duration of 20 min. The best preventive measure is obviously an adjusted FFP2 mask (case FF) for both infectious and susceptible, and the least safe measure is for both to wear a surgical mask (case SS). Interestingly, very loosely fitted FFP2 masks (case ff) outperform adjusted surgical masks (case SS) by a factor of 2.5. Proper nosepiece adjustment for FFP2 masks can decrease risk of infection by a factor of 30 (case FF vs. case ff), while, if at least one of the infectious or susceptible adjust their FFP2 masks, the increase in risk compared to case FF is about 5 to 7 times. Risk of infection for asymmetric cases, that is, Ff vs. fF, FS vs. SF, and fS vs. Sf, is lower by about 7 to 50% when the better mask or the better-adjusted mask is worn by the infectious. This suggests that the masks are more effective outwardly (protection of third parties). As shown in *SI Appendix*, section 2.H, the combined penetration resulting from TIL×TOL is such that the risk of infection is lower when the better mask is worn by the infectious. Nevertheless, the risk of infection for some of the combinations is too close to represent a significant difference. Slight variations in the assumptions considered, for example, when TOL≠TIL, might change the order in Fig. 6 slightly. However, the absolute magnitude would remain a true upper bound because we used the extremely conservative value of $f_{d}=1.0$. Considering all these points, the safe conclusions from Fig. 6 are the following: 1) the use of a well-fitting FFP2 mask by both individuals, case FF, is the best combination, 2) the use of a well-fitting FFP2 mask by either the infectious or the susceptible, cases Ff, fF, FS, and SF, is the next best measure, 3) a loosely worn FFP2 mask by both or by one of the individuals (cases ff, fS, and Sf) is likely to provide better protection than if both wear a well-fitting surgical mask (case SS), and 4) the overall risk of infection, regardless of mask, is very low if all adhere to masking, as even the highest upper bound with extremely conservative input parameters yields a risk of infection of only 10%.

We have also investigated whether there is any significant difference between the polypathogen and monopathogen models, and found that, for the scenarios considered here, the differences are negligible. It is expected, in scenarios with higher viral loads and for activities associated with production of larger particles, for example, sneezing, that the differences become more significant.

## Conclusion

We have calculated an upper bound on the exposure/risk of infection between two individuals in the near-field and short ( <1 h) exposure duration with different masking and social distancing scenarios using typical estimates of SARS-CoV-2 viral load and infectious dose. We used a comprehensive database of human exhaled particles, the fluid dynamics of exhalation jets, and the measured leakage from face masks of various types and fits on human subjects. In calculating the risk of infection, we also considered particle shrinkage in the environment due to evaporation, as well as rehydration, inhalability, and deposition in the airways of the susceptible as a function of particle size. The risk was calculated with the polypathogen model, although the results are similar for a monopathogen exponential model.

With the aim of calculating an upper bound of infection risk when a susceptible is exposed to an infectious in near field, we have considered three scenarios. When both infectious and susceptible wear face mask (mask scenario), we have assumed the infectious is exposed to an undiluted respiratory particle concentration penetrated through the fabric and leaks of the infectious’ mask, that is, particle loss due to wearing a mask being the only particle removal mechanism. When the infectious is not wearing a mask, we have assumed the unmasked susceptible (distancing scenario) or the masked susceptible (mixed scenario) is at a fixed distance from the infectious and always in the infectious’ exhalation cone. In this case, the concentration to which the susceptible is exposed has an inverse relationship with the distance from infectious due to turbulent dilution alone (particle loss due to deposition on the ground not considered). For all the investigated scenarios, we have also assumed particle diameter to shrink by a factor of 4 before reaching the susceptible (independent of the distance between infectious and susceptible) and pathogen inactivation being negligible.

The upper bound presented here is, by definition, extremely conservative, especially for the mask scenario. Therein lies the true strength of the results presented, as they are based on the smallest possible number of parameters. The upper bounds do not need to address the details of the specific situation, which include the long list of parameters we have already mentioned for the source–medium–receptor trilogy. We have therefore intentionally not given a specific value for the overestimation, as this can range from no overestimation to a factor of several thousand, depending on the situation. Thus, if a scenario proves to be safe under the upper bounds defined here, the question of its effectiveness under real-world conditions does not arise. Of course, the concept of upper bounds also applies to the relationship between exposure duration and infection risk, which is also situation dependent. In all cases, the upper bounds allow a simple comparison between the different scenarios considered.

Our results show that social distancing alone without masking is associated with a very high risk of infection, especially in situations where infectious is speaking. High infection risks are also expected when only the susceptible wears a face mask, even with social distancing. We show that universal masking is the most effective method for limiting airborne transmission of SARS-CoV-2, even when face seal leaks are considered. The main factor affecting infection risk in the universal masking scenario is leakage between the mask and the face. The fitted FFP2 masks studied here (and, most likely, other vertically folded FFP2 masks of similar design), when properly fitted to infectious and susceptible faces, can reduce the risk of infection by a factor of 30 compared with loosely worn masks and by a factor of 75 compared with fitted surgical masks for an exposure duration of 20 min. Our results also suggest that the use of FFP2 masks should be preferred to surgical masks, as even loosely worn FFP2 masks can reduce the risk of infection by a factor of 2.5 compared with well-fitted surgical masks. Considering that the upper bound for infection risk used here is, by definition, extremely conservative, we conclude that universal masking with surgical masks and/or FFP2 masks is a very effective measure to minimize the transmission of COVID-19.

## Materials and Methods

### Mask Efficacy Measurements

#### Filter penetration

The details of the methods of the filter penetration are described in *SI Appendix*, section 1.C.

#### Total inward leakage

Information about dimensions and surface area of masks are presented in *SI Appendix*, section 1.B. A drawing of the measurement setup is shown in *SI Appendix*, Fig. S3. The total inward leakage *TIL* of a face mask is defined as the ratio of particle concentration inside the mask, *c_mask_*, to particle concentration in the background, *c_bg_*, that is, $\mathrm{TIL}=c_{\mathrm{mask}}/c_{bg}$. The *TIL* is also equivalent to the weighted sum of the inward filter penetration *P_in_*  =  *P_filter_* and inward particle penetration through face seal leaks *L_in_*, hence, $\mathrm{TIL}=q_{P,in}P_{in}+q_{L,in}L_{in}$. To measure particle size–dependent total inward leakage from the surgical and FFP2 masks with different fittings, worn separately or together, we have investigated several combinations of masks/fittings (also see the legend of Fig. 2):

1) case *i*: FFP2 mask without adjustment/shaping the nosepiece (also called nose clip or nose wire) at all, which was delivered folded and, hence, with a very sharp bend;
2) case *ii*: vertical-fold FFP2 mask fully adjusted to the face by reshaping the nose bridge, avoiding any sharp bend above the nose bridge and also shaped on the sides (i.e., in the transition area from the nose to the cheek), and pressing the nose bridge firmly with two fingers moving simultaneously from above the nose to the sides;
3) case *iii*: FFP2 mask with a surgical mask over it, both with adjustment of the nosepiece fitting by prebending;
4) case *iv*: FFP2 mask with a 1× 12 cm double-sided 3M Medical Tape 1509 applied underneath the nosepiece to fully seal the mask to the nose and areas around it; and
5) case *v*: surgical mask with adjustments to the nosepiece fitting similar to case *ii*.

These cases have been measured on seven adult subjects (one female and six males, three of whom had noticeable facial hair; subjects’ facial dimensions are presented in *SI Appendix*, section 1.A and Table S1) while they were breathing normally through the nose. The experiments were performed in a 200-m^3^ room. Before starting measurements, the test particles (dolomite dust from DMT GmbH & Co KG) were released into the room by shaking a dust-covered microfiber cloth in front of a 120-W fan with 0.26-m blades, to mix the particles in the room air. The fan was running throughout the measurements while it was 3 m away from the subject and oriented at an angle of ∼30^∘^ toward the ceiling to reduce potential nonisokinetic sampling bias in the measurement of background concentrations. New particles were released into the room periodically to compensate for the loss of large particles in the background due to deposition on the floor and fan blades. The total inward leakage of the different cases *i* to *v* were examined for each subject while they were seated in a chair. Subjects were in a relaxed sitting position for at least 1 min before measurements began. Two OPS spectrometers (see *Materials and Methods*) were used synchronously to measure the background and inhaled air samples simultaneously. A sampling resolution of 1 s was chosen, and total inward leakage was measured for a duration of at least 100 s for each case (a total duration of ∼15 min per subject). The sampling flow rate of both spectrometers was 1 L×min^−1^. The sampling tube measuring in-mask concentrations *c_mask_* was held in place by an easily adjustable arm attached to a helmet worn by the subjects as shown in *SI Appendix*, Fig. S3. The in-mask sampling tube was connected to a plastic feed-through that passed through a punched hole in the mask with a diameter of 8 mm and was tightened onto the mask from the inside with a nut. The horizontal position of the feedthrough was 1 cm from the middle seam of the mask, and the vertical position was chosen for each subject individually so that it was half-way between the nose and upper lip. Test experiments with different locations showed that the chosen inlet position did not lead to significantly different results than a lower inlet position between the lower lip and chin (*SI Appendix*, Fig. S7). The helmet arm for holding the inhalation sampling tube was adjusted for each subject so that the tube neither pressed on nor pulled on the mask. Moreover, the last 35 cm of the sampling tube was made out of a more flexible rubber material compared to the conductive polytetrafluorethylene (PTFE) tube to minimize any forcing on the mask. The background concentration *c_bg_* was measured ∼20 cm in front of the subject’s head. Both sampling tubes (in-mask and background) had the exact same lengths and approximately the same curvatures. In any case, possible differences between the particle paths and OPSs were corrected by performing sensitivity corrections as described in *SI Appendix*, section 1.E.1. Furthermore, each sampling tube was connected to a diffusion dryer (TOPAS DDU570/H) to remove humidity that could alter measured particle sizes (see *SI Appendix*, Fig. S8 for comparison against measurements without diffusion dryer). All tubes and connections were checked for potential leaks before measurements by using a HEPA filter and observing zero particle counts on the spectrometers.

#### Mask data analysis

To correct for varying sensitivities in the different bins of the spectrometers (OPS and SMPS), a running geometric average over three bins each was used. For the leakage measurements that were performed with two OPS spectrometers simultaneously, the possibly varying sensitivities and different particle loss rates inside the tubes and diffusion dryers were corrected with a 27-min-long simultaneous calibration background measurement. More details can be found in *SI Appendix*, section 1.E.1. For the total inward leakage calculation, only the in-mask particle concentration during inhalation should be included in the analysis (70). Inhalation is associated with a peak in in-mask particle concentration. Therefore, only samples within the top 10% of a peak in total particle count were considered for the total inward leakage analysis. More details on this can be found in *SI Appendix*, section 1.E.2.

#### Total outward leakage

The total outward leakage *TOL* is the sum of outward filter penetration *P_ex_* and the outward face seal leakage during exhalation *L_ex_*, that is, $\mathrm{TOL}=q_{P,ex}P_{ex}+q_{L,ex}L_{ex}$. Filter penetration should not depend on flow direction, and, therefore, $P_{ex}=P_{in}=P_{\mathrm{filter}}$. Outward leakage of masks was not measured in this study. Previous studies on outward leakage paint an inconclusive picture. Van der Sande et al. (46) found larger total outward leakage (smaller outward protection factors) for an FFP2 and surgical mask compared to total inward leakage. The difference is more significant for the FFP2 mask (ratio TOL/TIL up to ∼50 for FFP2 and ∼2 for surgical masks). This could be explained by a high-pressure difference between inside and outside the mask which pushes the mask away from the face, leading to higher leakage (71). However, in their study, total inward leakage was measured on human subjects, whereas total outward leakage was studied on a manikin, which could influence mask fit. In contrast, a pure manikin study by Koh et al. (72) found no difference between inward and outward leakage for a fitted N95 respirator. For an unfitted N95 mask and a surgical mask, outward leakage was measured to be slightly lower than inward leakage, which was also observed in another manikin study by Pan et al. (73) for a surgical mask. In ref. 73, the difference between inward and outward leakage was found to vary from mask to mask. For the surgical mask, inward leakage was also larger than outward leakage. The ratio TOL/TIL ranged between 0.5 and 1 in those studies. These inconclusive results combined with uncertainties in the experimental methods lead us to assume $L_{in}\approx L_{ex}$ and therefore TOL≈TIL.

### Infection Risk Model

Considering the fact that exhaled particles can contain one or more pathogens, depending on their size and pathogen concentration, the average infection probability, hereafter referred to as infection risk, can be calculated via ([3])

[2] $$R_{I}=1-\exp\;\left[-\sum_{k=1}^{\infty }\left(1-{\left(1-r\right)}^{k}\right)\mu_{k}\right]\;,$$

where *μ_k_* is the absorbed aerosol dose with multiplicity of *k*, that is, containing *k* pathogens inside, and *r* is the probability of each pathogen causing infection. Note that r=1/D, where *D* is the infectious dose required for 63.21% chance of infection, or ID${}_{63.21}$. Details on how to calculate the absorbed aerosol dose are given in ref. 3. In the traditional exponential model, only particles with *k* = 1 are considered, in which case Eq. **2** reduces to $R_{I,\mathrm{mono}}=1-e^{-r\;\mu_{1}}$, where *μ*_1_ is the average number of monopathogen-borne particles absorbed. The monopathogen infection risk $R_{I,\mathrm{mono}}$ always overestimates the infection risk compared to the polypathogen *R_I_* formulation shown in Eq. **2** (see ref. 3 for more details). In order to calculate the infection risk and the average absorbed dose, the following assumptions are made:

• initial pathogen concentration in the room and initial absorbed pathogen dose of the susceptible are both zero;
• airborne transmission from only one infectious to only one susceptible is considered;
• susceptible is always in the exhale cone of the infectious;
• pathogen accumulation in the ambient is negligible, and, thus, susceptible can inhale pathogens only when the infectious is active; as a result, exposure duration is smaller than or equal to source-active duration; and
• exposure duration is much shorter than the time needed for the pathogen inactivation to be significant.

Given these assumptions, the total absorbed dose by the susceptible individual can be calculated as follows:

[3] $$\begin{array}{l} \mu_{k}(t)=\int_{d_{0,\min}(k)}^{d_{0,\max}(k)}\;\text{d}\phi \int_{0}^{t_{\exp}}\text{d}t\\ \times \overset{\text{infec}.\;\text{particle}\;\text{conc}.\;\text{in}\;\text{breath}.\;\text{zone}\;\text{of}\;\text{susceptible}}{\overset{︷}{n_{I,k}(\phi ,t)\;f_{d}\left(\phi ,\lambda_{I}(t),w(\phi ,t),t\right)}}\\ \times \overset{\text{total}\;\text{outward}\;\text{leakage}\;(\mathrm{TOL})}{\overset{︷}{\left[q_{P,ex}P_{ex}(\phi ,\lambda_{I}(t))+q_{L,ex}L_{ex}(\phi ,\lambda_{I}(t))\right]}}\\ \times \overset{\text{total}\;\text{inward}\;\text{leakage}\;(\mathrm{TIL})}{\overset{︷}{\left[q_{P,in}P_{in}(\phi ,w(\phi ,t),\lambda_{S}(t))+q_{L,in}L_{in}(\phi ,w(\phi ,t),\lambda_{S}(t))\right]}}\\ \times \overset{\;\;\;\;\text{intake\&deposition}\;\text{eff}.\;\text{susc}.\;\text{resp}.\;\text{tract}}{\overset{︷}{D_{rt}(\phi ,w(\phi ,t),\lambda_{S}(t))}}\;\overset{\;\;\;\;\text{susc}.\;\text{inhalation}\;\text{rate}}{\overset{︷}{\lambda_{\text{S}}(\text{t})}}\;, \end{array}$$

where

[4] $$n_{I,k}(d_{0},t)=\{\begin{array}{ll} C_{n,I}\left(d_{0},t\right)e^{-\langle k\rangle \text{​}\left(d_{0}\right)}\left(\frac{\langle k\rangle \text{​}{\left(d_{0}\right)}^{k}}{k!}\right) & \text{if}\;d_{0}\geq d_{0,\min}(k)\;,\\ 0 & \text{if}\;d_{0}<d_{0,\min}(k)\;, \end{array}$$

and $\langle k\rangle \text{​}\left(d_{0}\right)=(\pi /6)d_{0}^{3}\rho_{p}$, *d*_0_ is the initial wet particle size on exhalation by the infectious, $w(d_{0},t)=d_{0}/d_{e}$ is the shrinkage factor defined as the ratio of the particle initial wet diameter *d*_0_ to the equilibrium diameter *d_e_* after it is exposed to the typically subsaturated conditions of the room and has lost its volatile components, $d_{0,\min}(k)$ and $d_{0,\max}(k)$ are the minimum and maximum particle size that can be aerosolized and contain *k* copies of the pathogen, *t_exp_* is the exposure duration of the susceptible individual, *ρ_p_* is the pathogen number concentration, that is, the viral load, in the infectious respiratory tract fluid, $C_{n,I}(d_{0},t)$ is the number concentration of exhaled particles at the mouth/nose of the infectious, $f_{d}(d_{0},\lambda_{I}(t),w(d_{0},t),t)$ is the fractional ratio at which the particle concentration of the exhaled air by the infectious individual decreases until it reaches the breathing zone of the susceptible individual due to (turbulent and/or molecular) mixing with the room air or particle deposition losses, $P_{ex}(d_{0},\lambda_{I})$ is the outward filter penetration of the face mask fabric worn by the infectious, $L_{ex}(d_{0},\lambda_{I})$ is the outward face seal leakage of the face mask worn by the infected individual during exhalation, $q_{P,ex}$ and $q_{L,ex}$ are, respectively, the ratios of the exhale flow rate through the filter and face seal leaks to the total exhale flow rate of the infectious, $P_{in}(d_{0},w(d_{0},t),\lambda_{S})$ is the inward filter penetration of the face mask fabric worn by the susceptible, $L_{in}(d_{0},w(d_{0},t),\lambda_{S})$ is the inward face seal leakage of the face mask worn by the susceptible, $q_{P,in}$ and $q_{L,in}$ are, respectively, the ratios of the inhale flow rate through the filter and face seal leaks to the total inhale flow rate of the susceptible, $D_{rt}(d_{0},w(d_{0},t),\lambda_{S}(t))$ is the intake/deposition efficiency of the inhaled particles within the respiratory of the susceptible individual, and $\lambda_{I}(t)$ and $\lambda_{S}(t)$ are the volumetric inhalation rate (also called ventilation rate) of the infectious and susceptible, respectively. It should be noted that many of the parameters present in Eq. **3** are also functions of the room conditions, for example, RH, temperature, ventilation type, air velocity, which are neglected here.

We assume that the *ρ_p_* is constant and independent of the particle size, even though it has been shown that particles of different sizes have different production sites within the respiratory tract (5) and particles of different origins might have different viral loads (74). The SARS-CoV-2 viral load, *ρ_p_*, is in the very broad range of 10^2^mL^−1^ to 10^11^ mL^−1^ (23). Mean values for the currently measured SARS-CoV-2 variants are 10^8.2^ mL^−1^ to 10^8.5^ mL^−1^ (75). Here we use 10^8.5^ mL^−1^ to obtain an upper estimate on risk of infection, which should be more applicable to the new variants of SARS-CoV-2. The increase in viral load with the new variants currently circulating globally is constant with findings in other studies (e.g., see ref. 76, and references therein). The SARS-CoV-2 ID${}_{63.21}$ is not known very well, and, in the literature, a range of values between 100 and 1,000 is used, that is, ∼400 (3, 21) and 100 (26). In this investigation, we assume ID${}_{63.21}=200$, which, for a fixed pathogen dose, gives a risk of infection that is, at most, half (or 2 times) the values calculated with ID${}_{63.21}=100$ (or 400). $C_{n,I}$ values are calculated based on the multimodal fits found by ref. 5, which is obtained based on measurements from more than 130 subjects aged 5 y to 80 y, using aerosol size spectrometers and in-line holography covering wet particle sizes, that is, *d*_0_, from 50 nm up to 1 mm. The multimodal fits presented by ref. 5 provide an average estimation of $C_{n,I}$ for an adult (gender plays no role). The smallest particle size considered for infection risk analyses, that is, $d_{0,\min}$, is 0.2 μm, which is about 2 times the size of the SARS-CoV-2 virus (e.g., see refs. 3 and 4). As for the upper limit, we considered $d_{0,\max}=50\mu \text{m}$ and assumed larger particles deposit to the ground very quickly and in the vicinity of the infectious person. However, it should be noted that there is an ongoing debate regarding the advection distance of exhaled particles in different respiratory activities and room conditions (e.g., see refs. 9 and 29, and references therein, for more details). Particles exhaled by the infectious are moist and, depending on the RH, may decrease considerably in size by evaporation until they reach the breathing zone of the susceptible. Unless otherwise stated, we have assumed all the particles shrink by a factor of 4, that is, *w* = 4.0, which is the expected shrinkage factor for RH<30% (5), which is a conservative estimate for RH encountered in typical indoor environments (4). The values published in table 15 of ref. 53 are used to calculate $\lambda_{I}(t)$ and $\lambda_{S}(t)$. However, since these rates are given for general physical activities, that is, sleeping, sitting, and light and heavy exercise, they are combined by optimal weighting factors that were found iteratively and that reproduced the rates found in the literature for different respiratory activities (77–79). Breathing and speaking ventilation rates assumed to be constant and equal to 0.57 m^3^×h^−1^ and 0.67 m^3^×h ^−1^, respectively. While *P_ex_* and *L_ex_* are functions of particles diameter during exhalation, *d*_0_, *P_in_*, and *L_in_* are dependent on particle diameter during inhalation, $d_{e}=d_{0}/w$. The penetration of mask fabric is also a function of breathing rates since it will influence the particle loss due to inertial impaction (important for larger than 1-μm particles) and the time required for capturing submicron particles due to Brownian diffusion. The penetration due to mask leakage is also a function of particle diameter and breathing rate; more details regarding these parameters can be found in *Mask Efficacy Measurements*. The ICRP respiratory tract deposition (ICRP94) model (53) is used to calculate $D_{rt}(d_{0},w(d_{0},t),\lambda_{S}(t))$, The ICRP94 model can provide an estimate of particle inhalability and also the deposition efficiency in five different regions of the respiratory tract based on empirical and numerical models, namely, nasal, oral, thoracic bronchial, bronchioles, and alveolar regions. In order to capture the deposition of exhaled particles that have dried in the typically subsaturated air of a room, one also needs to consider that such particles will undergo hygroscopic growth as they enter the almost saturated environment within the respiratory tract, that is, with an RH of 99.5% (4, 53, 80, 81). To take into account the hygroscopic growth of inhaled particles, the coupled equations describing rate of change in the particle size and its temperature are solved simultaneously, as explained well in section 13.2.1 of ref. 82, assuming fully dried particles consisting of pure NaCl crystals. This assumption is a good approximation for human aerosols, although a more detailed knowledge would be highly beneficial. The osmotic coefficient required for hygroscopic growth of the NaCl solution is calculated via formulations provided by ref. 83. The hygroscopic growth codes are verified against diffusional growth rate curves shown in figure 13.2 of ref. 82 and also those produced by the E-AIM web-app (84). For all regions, the midresidence time in the region plus the time spent in all the previous regions is taken as the time duration for calculating the grown size of particles. The total time duration that the particles spend in the respiratory tract per each inhalation+exhalation maneuver is calculated as $60/f_{R}$, where *f_R_* is the respiration frequency per minute provided by the ICRP94 model. The time that particles spend in each region is then calculated by the distribution of the total respiration time according to the time constants provided by the ICRP94 deposition model for thoracic bronchial, bronchioles, and alveolar regions. The particle residence times for the extrathoracic regions, which are not provided in the ICRP94 model, during inhalation or exhalation are assumed to be 0.1 s. The susceptible is assumed to be a 35-y-old nose-breather male. As mentioned above, the fractional ratio $f_{d}\left(d_{0},\lambda_{I}(t),w(d_{0},t),t\right)$ is one of the most challenging parameters in Eq. **3**. Even the most detailed simulations to date are carried out by assuming the exhale flow behaves similarly to a turbulent jet in a room with quiescent air (e.g., see refs. 9 and 29, and references therein). Therefore, for situations where the infectious is not wearing a face mask, we use a simplified theoretical formulation recently proposed for particle-laden jet flows (26, 27), that is, $f_{d}=a/(x\;\tan(\alpha ))$, where *x* is the distance between the source and the receptor, *a* is the radius of the mouth (assuming a circular shape), and *α* is the exhale jet half-angle. For x= 1 m, a= 1.2 cm, and α≈ 10^∘^, *f_d_* is ∼6.8%, which agrees well with the 4.9% experimentally measured for 0.77-μm particles by ref. 85. For nose breathing, ref. 79 found an average nose opening area of 0.56 cm^2^ to 0.71 cm^2^ (a = 0.42 cm to 0.48 cm) and α≈ 11.5^∘^, where $f_{d}=$ 2 to 3% at a distance of 1 m. For mouth breathing, ref. 79 found a≈ 0.61 cm to 0.75 cm and α≈ 17^∘^, where $f_{d}=2\%$ at a distance of 1 m. For speaking, ref. 79 found an average mouth opening of 1.8 cm^2^, which corresponds to a≈ 0.76 cm; however, no information for *α* is presented. In order to be on the conservative side when calculating infection risk, we assume a= 1.8 cm and α= 10^∘^ to achieve $f_{d}=0.1$ at a distance of 1 m. These values are used for all scenarios in which the infectious is not wearing a face mask, to calculate *f_d_*. For scenarios in which the infectious is wearing a face mask, *f_d_*  =  1.

## Supplementary Material

Supplementary File

## Data Availability

Previously published data were used for this work (https://aerosol.ds.mpg.de/). All other study data are included in the article and/or *SI Appendix*.

## References

[1] R. Zhang, Y. Li, A. L. Zhang, Y. Wang, M. J. Molina, Identifying airborne transmission as the dominant route for the spread of COVID-19. Proc. Natl. Acad. Sci. U.S.A. 117, 14857–14863 (2020). 32527856 10.1073/pnas.2009637117 PMC7334447

[2] WHO Team, Transmission of SARS-CoV-2: Implications for infection prevention precautions. (Sci. Brief, World Health Organization, 2020). https://www.who.int/news-room/commentaries/detail/transmission-of-sars-cov-2-implications-for-infection-prevention-precautions. Accessed 24 November 2021.

[3] F. Nordsiek, E. Bodenschatz, G. Bagheri, Risk assessment for airborne disease transmission by poly-pathogen aerosols. PLoS One 16, e0248004 (2021). 33831003 10.1371/journal.pone.0248004 PMC8031403

[4] M. L. Pöhlker ., Respiratory aerosols and droplets in the transmission of infectious diseases. *arXiv* [Preprint] (2021). https://arxiv.org/abs/2103.01188 (Accessed 4 August 2021).

[5] G. Bagheri ., Exhaled particles from nanometre to millimetre and their origin in the human respiratory tract. medRxiv [Preprint] (2021). 10.1101/2021.10.01.21264333 (Accessed 3 October 2021).

[6] T. Greenhalgh ., Ten scientific reasons in support of airborne transmission of SARS-CoV-2. Lancet 397, 1603–1605 (2021). 33865497 10.1016/S0140-6736(21)00869-2 PMC8049599

[7] N. H. L. Leung, Transmissibility and transmission of respiratory viruses. Nat. Rev. Microbiol. 19, 528–545 (2021). 33753932 10.1038/s41579-021-00535-6 PMC7982882

[8] K. Randall, E. T. Ewing, L. Marr, J. Jimenez, L. Bourouiba, How did we get here: What are droplets and aerosols and how far do they go? A historical perspective on the transmission of respiratory infectious diseases. Interface Focus 11, 20210049 (2021). 10.1098/rsfs.2021.0049 PMC8504878 34956601

[9] K. L. Chong ., Extended lifetime of respiratory droplets in a turbulent vapor puff and its implications on airborne disease transmission. Phys. Rev. Lett. 126, 034502 (2021). 33543958 10.1103/PhysRevLett.126.034502

[10] T. Greenhalgh, M. B. Schmid, T. Czypionka, D. Bassler, L. Gruer, Face masks for the public during the covid-19 crisis. BMJ 369, m1435 (2020). 32273267 10.1136/bmj.m1435

[11] D. K. Chu .; COVID-19 Systematic Urgent Review Group Effort (SURGE) study authors, Physical distancing, face masks, and eye protection to prevent person-to-person transmission of SARS-CoV-2 and COVID-19: A systematic review and meta-analysis. Lancet 395, 1973–1987 (2020). 32497510 10.1016/S0140-6736(20)31142-9 PMC7263814

[12] M. Z. Bazant, J. W. M. Bush, A guideline to limit indoor airborne transmission of COVID-19. Proc. Natl. Acad. Sci. U.S.A. 118, e2018995118 (2021). 33858987 10.1073/pnas.2018995118 PMC8092463

[13] C. J. Wang, C. Y. Ng, R. H. Brook, Response to COVID-19 in Taiwan. JAMA 323, 1341–1342 (2020). 32125371 10.1001/jama.2020.3151

[14] T. L. D. Huynh, The COVID-19 containment in Vietnam: What are we doing? J. Glob. Heal. 10, 010338 (2020). 10.7189/jogh.10.010338 PMC7182304 32373318

[15] S. Lim, H. I. Yoon, K. H. Song, E. S. Kim, H. B. Kim, Face masks and containment of COVID-19: Experience from South Korea. J. Hosp. Infect. 106, 206–207 (2020). 32540463 10.1016/j.jhin.2020.06.017 PMC7291980

[16] S. Y. S. Wong, K. O. Kwok, F. K. L. Chan, What can countries learn from Hong Kong’s response to the COVID-19 pandemic? CMAJ 192, E511–E515 (2020). 32332040 10.1503/cmaj.200563 PMC7234274

[17] N. H. L. Leung ., Respiratory virus shedding in exhaled breath and efficacy of face masks. Nat. Med. 26, 676–680 (2020). 32371934 10.1038/s41591-020-0843-2 PMC8238571

[18] J. Howard ., An evidence review of face masks against COVID-19. Proc. Natl. Acad. Sci. U.S.A. 118, e2014564118 (2021). 33431650 10.1073/pnas.2014564118 PMC7848583

[19] T. Mitze, R. Kosfeld, J. Rode, K. Wälde, Face masks considerably reduce COVID-19 cases in Germany. Proc. Natl. Acad. Sci. U.S.A. 117, 32293–32301 (2020). 33273115 10.1073/pnas.2015954117 PMC7768737

[20] J. T. Brooks, J. C. Butler, Effectiveness of mask wearing to control community spread of SARS-CoV-2. JAMA 325, 998–999 (2021). 33566056 10.1001/jama.2021.1505 PMC8892938

[21] J. Lelieveld ., Model calculations of aerosol transmission and infection risk of COVID-19 in indoor environments. Int. J. Environ. Res. Public Health 17, 8114 (2020). 10.3390/ijerph17218114 PMC7662582 33153155

[22] G. Buonanno, L. Stabile, L. Morawska, Estimation of airborne viral emission: Quanta emission rate of SARS-CoV-2 for infection risk assessment. Environ. Int. 141, 105794 (2020). 32416374 10.1016/j.envint.2020.105794 PMC7211635

[23] S. L. Miller ., Transmission of SARS-CoV-2 by inhalation of respiratory aerosol in the Skagit Valley Chorale superspreading event. Indoor Air 31, 314–323 (2021). 32979298 10.1111/ina.12751 PMC7537089

[24] J. L. Jimenez, COVID-19 Aerosol Transmission Estimator (Version 3.4.19 released 2020 Oct. 27). https://tinyurl.com/covid-estimator. Accessed 26 November 2020.

[25] Max Plank Institut for Dynamics and Self-Organization, HEADS – Human Emission of Aerosol and Droplet Statistics. https://aerosol.ds.mpg.de. Accessed 4 May 2021.

[26] F. Yang, A. A. Pahlavan, S. Mendez, M. Abkarian, H. A. Stone, Towards improved social distancing guidelines: Space and time dependence of virus transmission from speech-driven aerosol transport between two individuals. Phys. Rev. Fluids 5, 122501 (2020).

[27] M. Abkarian, S. Mendez, N. Xue, F. Yang, H. A. Stone, Speech can produce jet-like transport relevant to asymptomatic spreading of virus. Proc. Natl. Acad. Sci. U.S.A. 117, 25237–25245 (2020). 32978297 10.1073/pnas.2012156117 PMC7568291

[28] Y. Cheng ., Face masks effectively limit the probability of SARS-CoV-2 transmission. Science 372, 1439–1443 (2021). 10.1126/science.abg6296 PMC8168616 34016743

[29] J. Wang ., Short-range exposure to airborne virus transmission and current guidelines. Proc. Natl. Acad. Sci. U.S.A. 118, e2105279118 (2021). 34465564 10.1073/pnas.2105279118 PMC8449319

[30] W. C. Hinds, G. Kraske, Performance of dust respirators with facial seal leaks: I. Experimental. Am. Ind. Hyg. Assoc. J. 48, 836–841 (1987). 3687728 10.1080/15298668791385679

[31] W. R. Myers, H. Kim, N. Kadrichu, “Effect of particle size on respirator faceseal leakage” (Tech. Rep. AD-A235 439, West Virginia University, 1990).

[32] K. Willeke ., Filtration, loading and faceseal leakage characteristics of filtering facepieces. J. Aerosol Sci. 23, 749–752 (1992).

[33] A. Weber ., Aerosol penetration and leakage characteristics of masks used in the health care industry. Am. J. Infect. Control 21, 167–173 (1993). 8239046 10.1016/0196-6553(93)90027-2

[34] C. C. Chen, K. Willeke, Characteristics of face seal leakage in filtering facepieces. Am. Ind. Hyg. Assoc. J. 53, 533–539 (1992). 1524028 10.1080/15298669291360120

[35] K. J. Cho ., Large particle penetration through N95 respirator filters and facepiece leaks with cyclic flow. Ann. Occup. Hyg. 54, 68–77 (2010). 19700488 10.1093/annhyg/mep062 PMC6768069

[36] S. Rengasamy, B. C. Eimer, Total inward leakage of nanoparticles through filtering facepiece respirators. Ann. Occup. Hyg. 55, 253–263 (2011). 21292731 10.1093/annhyg/meq096

[37] S. Rengasamy, B. C. Eimer, Nanoparticle penetration through filter media and leakage through face seal interface of N95 filtering facepiece respirators. Ann. Occup. Hyg. 56, 568–580 (2012). 22294504 10.1093/annhyg/mer122

[38] S. Rengasamy, B. C. Eimer, J. Szalajda, A quantitative assessment of the total inward leakage of NaCl aerosol representing submicron-size bioaerosol through N95 filtering facepiece respirators and surgical masks. J. Occup. Environ. Hyg. 11, 388–396 (2014). 24275016 10.1080/15459624.2013.866715 PMC4589201

[39] X. He, T. Reponen, R. T. McKay, S. A. Grinshpun, Effect of particle size on the performance of an N95 filtering facepiece respirator and a surgical mask at various breathing conditions. Aerosol Sci. Technol. 47, 1180–1187 (2013). 31548759 10.1080/02786826.2013.829209 PMC6756464

[40] W. C. Hill, M. S. Hull, R. I. MacCuspie, Testing of commercial masks and respirators and cotton mask insert materials using SARS-CoV-2 virion-sized particulates: Comparison of ideal aerosol filtration efficiency versus fitted filtration efficiency. Nano Lett. 20, 7642–7647 (2020). 32986441 10.1021/acs.nanolett.0c03182 PMC7534799

[41] L. E. Bowen, Does that face mask really protect you? Appl. Biosaf. 15, 67–71 (2010).

[42] U. Pauli, S. Karlen, K. Summermatter, The importance of fit-testing particulate filtering facepiece respirators! Appl. Biosaf. 19, 184–192 (2014).

[43] R. B. Lawrence, M. G. Duling, C. A. Calvert, C. C. Coffey, Comparison of performance of three different types of respiratory protection devices. J. Occup. Environ. Hyg. 3, 465–474 (2006). 16857645 10.1080/15459620600829211

[44] A. C. Lai, C. K. Poon, A. C. Cheung, Effectiveness of facemasks to reduce exposure hazards for airborne infections among general populations. J. R. Soc. Interface 9, 938–948 (2012). 21937487 10.1098/rsif.2011.0537 PMC3306645

[45] S. Rengasamy ., Total inward leakage measurement of particulates for N95 filtering facepiece respirators–A comparison study. Ann. Occup. Hyg. 58, 206–216 (2014). 24107745 10.1093/annhyg/met054

[46] M. van der Sande, P. Teunis, R. Sabel, Professional and home-made face masks reduce exposure to respiratory infections among the general population. PLoS One 3, e2618 (2008). 18612429 10.1371/journal.pone.0002618 PMC2440799

[47] T. Oberg, L. M. Brosseau, Surgical mask filter and fit performance. Am. J. Infect. Control 36, 276–282 (2008). 18455048 10.1016/j.ajic.2007.07.008 PMC7115281

[48] J. Gawn, M. Clayton, C. Makison, B. Crook, “*Evaluating the protection afforded by surgical masks against influenza bioaerosols*” (Res. Rep. 619, Health and Safety Executive, 2008).

[49] S. A. Lee ., Respiratory protection provided by N95 filtering facepiece respirators against airborne dust and microorganisms in agricultural farms. J. Occup. Environ. Hyg. 2, 577–585 (2005). 16234218 10.1080/15459620500330583

[50] S. A. Lee, S. A. Grinshpun, T. Reponen, Respiratory performance offered by N95 respirators and surgical masks: Human subject evaluation with NaCl aerosol representing bacterial and viral particle size range. Ann. Occup. Hyg. 52, 177–185 (2008). 18326870 10.1093/annhyg/men005 PMC7539566

[51] S. A. Grinshpun ., Performance of an N95 filtering facepiece particulate respirator and a surgical mask during human breathing: Two pathways for particle penetration. J. Occup. Environ. Hyg. 6, 593–603 (2009). 19598054 10.1080/15459620903120086 PMC7196699

[52] S. A. Lee ., Particle size-selective assessment of protection of European standard FFP respirators and surgical masks against particles-tested with human subjects. J. Healthc. Eng. 2016, 8572493 (2016). 10.1155/2016/8572493 PMC5058571 27195721

[53] International Commission on Radiological Protection, Ed., Human Respiratory Tract Model for Radiology Protection: A Report of a Task Group of the International Commission on Radiological Protection (Pergamon, Oxford, UK, 1994). 7726471

[54] C. D. Zangmeister, J. G. Radney, E. P. Vicenzi, J. L. Weaver, Filtration efficiencies of nanoscale aerosol by cloth mask materials used to slow the spread of SARS-CoV-2. ACS Nano 14, 9188–9200 (2020). 32584542 10.1021/acsnano.0c05025

[55] F. G. Morais ., Filtration efficiency of a large set of COVID-19 face masks commonly used in Brazil. Aerosol Sci. Technol. 55, 1028–1041 (2021).

[56] A. Konda ., Aerosol filtration efficiency of common fabrics used in respiratory cloth masks. ACS Nano 14, 6339–6347 (2020). 32329337 10.1021/acsnano.0c03252

[57] F. Drewnick ., Aerosol filtration efficiency of household materials for homemade face masks: Influence of material properties, particle size, particle electrical charge, face velocity, and leaks. Aerosol Sci. Technol. 55, 63–79 (2021).

[58] A. Bałazy ., Do N95 respirators provide 95% protection level against airborne viruses, and how adequate are surgical masks? Am. J. Infect. Control 34, 51–57 (2006). 16490606 10.1016/j.ajic.2005.08.018

[59] N. Serfozo ., Size-resolved penetration of filtering materials from CE-marked filtering facepiece respirators. Aerosol Air Qual. Res. 17, 1305–1315 (2017).

[60] A. Bałazy ., Manikin-based performance evaluation of N95 filtering-facepiece respirators challenged with nanoparticles. Ann. Occup. Hyg. 50, 259–269 (2006). 16344291 10.1093/annhyg/mei058

[61] S. Rengasamy, B. C. Eimer, R. E. Shaffer, Comparison of nanoparticle filtration performance of NIOSH-approved and CE-marked particulate filtering facepiece respirators. Ann. Occup. Hyg. 53, 117–128 (2009). 19261695 10.1093/annhyg/men086

[62] European Committee for Standardization, “*Respiratory protective devices - Filtering half masks to protect against particles - Requirements, testing, marking*” (EN 149:2001+A1, BSI Standards, 2009).

[63] Z. Lei, J. Yang, Z. Zhuang, R. Roberge, Simulation and evaluation of respirator faceseal leaks using computational fluid dynamics and infrared imaging. Ann. Occup. Hyg. 57, 493–506 (2013). 23243192 10.1093/annhyg/mes085

[64] R. Perić, M. Perić, Analytical and numerical investigation of the airflow in face masks used for protection against COVID-19 virus–implications for mask design and usage. J. Appl. Fluid Mech. 13, 1911–1923 (2020).

[65] R. K. Oestenstad, H. K. Dillion, L. L. Perkins, Distribution of faceseal leak sites on a half-mask respirator and their association with facial dimensions. Am. Ind. Hyg. Assoc. J. 51, 285–290 (1990). 2346116 10.1080/15298669091369664

[66] R. K. Oestenstad, A. A. Bartolucci, Factors affecting the location and shape of face seal leak sites on half-mask respirators. J. Occup. Environ. Hyg. 7, 332–341 (2010). 20379896 10.1080/15459621003729909

[67] J. T. Mueller, S. Karimi, K. A. Poterack, M. T. A. Seville, S. M. Tipton, Surgical mask covering of N95 filtering facepiece respirators: The risk of increased leakage. Infect. Control Hosp. Epidemiol. 42, 627–628 (2021). 33557963 10.1017/ice.2021.50 PMC8047391

[68] W. C. Hinds, P. Bellin, Performance of dust respirators with facial seal leaks: II. Predictive model. Am. Ind. Hyg. Assoc. J. 48, 842–847 (1987). 3318364 10.1080/15298668791385688

[69] L. Bourouiba, E. Dehandschoewercker, J. W. M. Bush, Violent expiratory events: On coughing and sneezing. J. Fluid Mech. 745, 537–563 (2014).

[70] W. R. Myers, J. Allender, R. Plummer, T. Stobbe, Parameters that bias the measurement of airborne concentration within a respirator. Am. Ind. Hyg. Assoc. J. 47, 106–114 (1986). 3456696 10.1080/15298668691389423

[71] R. Mittal, R. Ni, J. H. Seo, The flow physics of COVID-19. J. Fluid Mech. 894, F2 (2020).

[72] X. Koh ., Outward and inward protections of different mask designs for different respiratory activities. medRxiv [Preprint] (2021). 10.1101/2021.04.07.21255097 (Accessed 30 April 2021).

[73] J. Pan, C. Harb, W. Leng, L. C. Marr, Inward and outward effectiveness of cloth masks, a surgical mask, and a face shield. Aerosol Sci. Technol. 55, 718–733 (2021).

[74] Y. J. Hou ., SARS-CoV-2 reverse genetics reveals a variable infection gradient in the respiratory tract. Cell 182, 429–446.e14 (2020). 32526206 10.1016/j.cell.2020.05.042 PMC7250779

[75] S. M. Kissler ., Densely sampled viral trajectories suggest longer duration of acute infection with B. 1.1. 7 variant relative to non-B. 1.1. 7 SARS-CoV-2. medRxiv [Preprint] (2021). 10.1101/2021.02.16.21251535 (Accessed 14 May 2021).

[76] T. C. Jones ., Estimating infectiousness throughout SARS-CoV-2 infection course. Science, 373, eabi5273 10.1126/science.abi5273 (2021). 34035154 PMC9267347

[77] J. D. Hoit, T. J. Hixon, P. J. Watson, W. J. Morgan, Speech breathing in children and adolescents. J. Speech Hear. Res. 33, 51–69 (1990). 2314085 10.1044/jshr.3301.51

[78] J. K. Gupta, C. H. Lin, Q. Chen, Flow dynamics and characterization of a cough. Indoor Air 19, 517–525 (2009). 19840145 10.1111/j.1600-0668.2009.00619.x

[79] J. K. Gupta, C. H. Lin, Q. Chen, Characterizing exhaled airflow from breathing and talking. Indoor Air 20, 31–39 (2010). 20028433 10.1111/j.1600-0668.2009.00623.x

[80] G. Ferron, S. Soderholm, Estimation of the times for evaporation of pure water droplets and for stabilization of salt solution particles. J. Aerosol Sci. 21, 415–429 (1990).

[81] D. M. Broday, P. G. Georgopoulos, Growth and deposition of hygroscopic particulate matter in the human lungs. Aerosol Sci. Technol. 34, 144–159 (2001).

[82] H. R. Pruppacher, J. D. Klett, Microstructure of Atmospheric Clouds and Precipitation (Springer, 2010).

[83] I. Tang, H. Munkelwitz, N. Wang, Water activity measurements with single suspended droplets: The NaCl-H2O and KCl-H2O systems. J. Colloid Interface Sci. 114, 409–415 (1986).

[84] A. S. Wexler, S. L. Clegg, Atmospheric aerosol models for systems including the ions H+, NH4+, Na+, SO42-, NO3-, Cl-, Br-, and H2O. J. Geophys. Res. Atmos. 107, ACH 14-1–ACH 14-14 (2002).

[85] S. Liu, A. Novoselac, Transport of airborne particles from an unobstructed cough Jet. Aerosol Sci. Technol. 48, 1183–1194 (2014).

