# Supplementary material for: An upper bound on one-to-one exposure to infectious human respiratory particles
Source: Proc Natl Acad Sci U S A. 2021 Dec 2;118(49):e2110117118. doi: 10.1073/pnas.2110117118 (PMC8670465; doi:10.1073/pnas.2110117118)
Supplement: Supplementary File [file pnas.2110117118.sapp.pdf]

PNAS
www.pnas.org

Supplementary Information for

An upper bound on one-to-one exposure to infectious human respiratory particles

Gholamhossein Bagheri, Birte Thiede, Bardia Hejazi, Oliver Schlenczek and Eberhard Bodenschatz

Gholamhossein Bagheri, Eberhard Bodenschatz.
E-mail: gholamhossein.bagheri@ds.mpg.de, eberhard.bodenschatz@ds.mpg.de

This PDF file includes:

Supplementary text
Figs. S1 to S20
Table S1
SI References

Supporting Information Text

1. Methods

A. Subjects. The mask leakage was examined with 7 subjects (6 male, 1 female). Three of the male subjects had no facial hair, the other three had a beard with a length between 5 and 20 mm. To characterize the facial dimensions, we used the menton-sellion length, the bizygomatic breadth, the depth from the zygomatic arch to the nose tip, and the lip length. These four dimension parameters are used in the EN149:2001+A1:2009 standard (1) and are listed in Table S1. An illustration of the dimension parameters and how they were measured is shown in Fig. S1.

Subject	Sex	Face length (mm)	Face breadth (mm)	Face depth (mm)	Lip length (mm)
1	M	123	146	101	52
2	M	123	145	114	52
3	M	114	130	104	56
4	M	121	141	99	55
5	M	124	149	116	60
6	F	108	138	97	52
7	M	116	138	102	51

Table S1. Overview of the subjects who participated in the mask leakage experiments. The face length was measured as the menton-sellion length, the face breadth is the bizygomatic breadth, the face depth is from the zygomatic arch to the nose tip, and the lip length is from cheilion to cheilion. The facial dimensions were taken with a sliding caliper and a ruler.

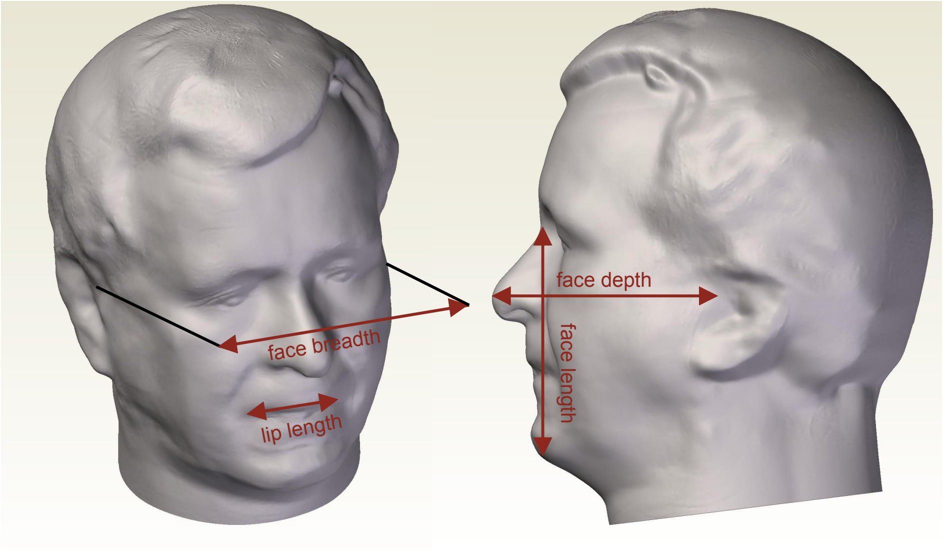

Fig. S1. Illustration of the facial dimensions used in Table S1. The left picture shows the face breadth (which is the bizygomatic breadth) and the lip length, the right picture shows the face length and face depth.

B. Masks. Figure S2 shows a schematic of the vertically folded FFP2 and folded surgical mask with their respective dimensions that were used in the leakage experiments. The nosepiece of the FFP2 mask is 8.4 cm long and 5 mm wide, whereas the one of the surgical mask is 10.6 cm long and 3 mm wide. The FFP2 mask has a penetrable surface of 233.3 cm^2, which was measured via the weight of the penetrable parts of the mask and the fabric area density. For the surgical mask, a rectangular penetrable surface with the inner width up to the seam and the inner unfolded height was assumed which leads to a penetrable surface of 214.9 cm^2.

C. Mask Filter Penetration. Two different setups were used to measure the particle size-dependent penetration through the filter material of different masks. In the first setup, hereafter referred to as "setup 1", three cloth, seven surgical and four FFP2 masks were tested. Each mask was fixed between two aluminum plates which were pressed together in a bench vice. An o-ring was used to ensure a good seal between the two metal plates and the mask. Both metal plates have a circular opening. The air can enter the mask from one side. The other side is connected to the sampling tube which leads via a Y-connection to the Optical Particle Sizer 3330 spectrometer (OPS) and the NanoScan Scanning Mobility Particle Sizer 3910 (SMPS) from TSI. With this the filtered particle concentration c_{filter} was measured. With the combination of OPS and SMPS, particles in the size range of 10 nm to 10 µm were counted in 13 + 16 logarithmically spaced bins. Since the OPS spectrometer has a sampling

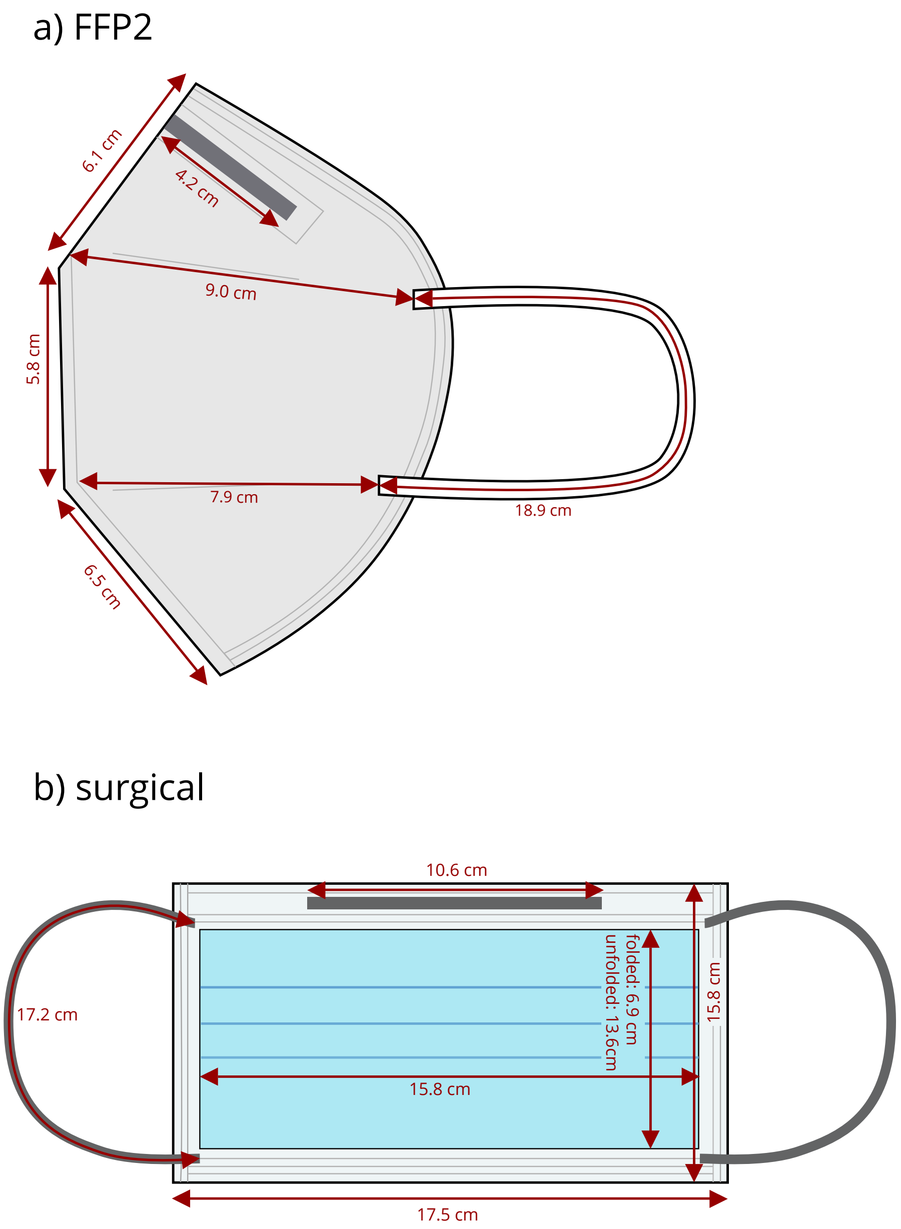

Fig. S2. FFP2 mask and surgical mask used in the mask leakage experiments. Uncertainties in length can be assumed to be $\pm 0.1\,\mathrm{cm}$. The elastic strings of the FFP2 mask are attached on the outside, whereas they are on the inside of the surgical mask.

flow rate of $1\,\mathrm{L\,min^{-1}}$ and the NanoScan SMPS of $0.76\,\mathrm{L\,min^{-1}}$, the total sampling flow rate was $1.76\,\mathrm{L\,min^{-1}}$ through the circular area with diameter of 3.2 cm. Applied to the whole penetrable mask surface of for example one FFP2 mask the flow velocities through the mask fabric in setup 1 ($3.7\,\mathrm{cm\,s^{-1}}$) are equivalent to those of a total flow rate of $51\,\mathrm{L\,min^{-1}}$ through the whole mask (similar for other masks as mask surface areas are comparable). The particles found naturally in the outdoor air were used as test particles. The background concentration c_{bg} was measured with the same combination of OPS and SMPS before and after each filter measurement. Both background and filter measurements were performed over at least 3 min each. The arithmetic mean of before and after measurements was used as the background for the penetration calculation. Please note that naturally occurring particles in outdoor air were found to be almost constant over time. The last bin of the SMPS was removed from the analysis as it overlaps with the first OPS bin which has better counting statistics by design. Particle diameters measured with the SMPS (below 0.3 µm) are electron mobility diameters whereas the OPS measures the optical diameter.

The second setup, hereafter referred to as "setup 2", was used to measure particle penetration through the filter material of masks used in the leakage experiments in this study (one type surgical and one type FFP2 mask, the masks' dimensions and designs are presented in the supplementary information, Fig. S2). Setup 2 is similar to setup 1 but the diameter of the circular opening in the metal plate was reduced to 1.6 cm. With this smaller opening we have a flow velocity of ($14.6\,\mathrm{cm\,s^{-1}}$) through the mask, equivalent to a total flow rate of $204\,\mathrm{L\,min^{-1}}$ through the whole FFP2 mask and $188\,\mathrm{L\,min^{-1}}$ through the whole surgical mask (which exceeds the $95\,\mathrm{L\,min^{-1}}$ required for FFP testing by the European standard). For setup 2, dolomite dust from DMT GmbH & Co KG that has particle size distribution of <20 µm was used as background particles in a well mixed closed room. For each mask, the filtered particle concentration was measured for 1 minute and the background was measured for 1 minute before and after each measurement. Since the background particle concentration exponentially decays, the geometric mean of the before and after background samples was used as the background concentration for the filter penetration calculation, where the filter penetration is defined as $P_{filter} = c_{filter}/c_{bg}$.

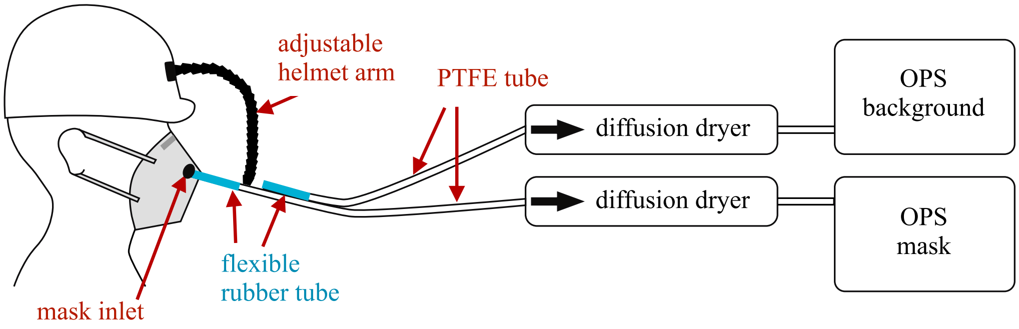

Fig. S3. Experimental setup for the total inward leakage measurements. The background air passes through a diffusion dryer and its particle size-dependent concentration is measured with an OPS. The in-mask sampling tube is held by an adjustable arm that is fixed to a helmet worn by the subject. It is sealed to the mask with a feedthrough. The air from inside the mask also passes a diffusion dryer and its particle size-dependent concentration is measured with a second OPS.

D. Final experimental setup. Figure S3 shows the experimental setup used in the final experiments to determine total inward leakage of seven different subjects as described in methods section A3 of the main paper.

E. Data analysis.

E.1. Sensitivity corrections. The sensitivity of each size bin in a single spectrometer cannot be assumed to be constant. To correct for this sensitivity bias in the particle size distribution the size-bin three-point running geometric average is used. This smoothing is applied to all raw OPS and SMPS particle counting data. The sensitivities of two different spectrometers for a specific bin can also differ. Moreover, the relatively long tubes and dryers used in the total inward leakage measurements are associated with particle losses that are highly size-dependent. Even though the same tubing setup was used for both spectrometers in the total inward leakage measurements, the particle losses can still be different. To account for both of these potential biases another correction was applied to the TIL data after smoothing over the size bins. This correction was obtained from a 27 min measurement where both spectrometers measured the same background concentration with the sampling tube endings right next to each other. The OPS spectrometer that was used in the background measurement was set as reference and the relative sensitivity of the in-mask measuring OPS was defined relative to it as $s_{OPSmask} = \frac{\langle c_{OPSmask} \rangle}{\langle c_{OPSbg} \rangle}$. The in-mask sampled data is then corrected before further analysis as $c_{in,corrected} = c_{in}/s_{OPSmask}$. The sampled particle counts for large particles are corrected by a higher factor, as they are more likely to get lost in tubes and dryers. Both methods together (the three-point running geometric average and the correction factor obtained from the instrument intercomparison) mostly correct both sensitivity biases. For the penetration experiments, a sensitivity correction between spectrometers was not necessary since the same spectrometers were used for filtered and background sampling.

E.2. Detection of inhalation periods. In the total inward leakage experiment, the particle concentration inside the mask was measured while the subject was breathing. It needs to be taken into account that the exhaled air is filtered by the lung and contains fewer particles. Analyzing the exhaled air would therefore bias our measurements of in-mask concentration negatively if not corrected for appropriately (2, eq. 5). To avoid this, we only use samples collected during inhalation as suggested by Myers *et al.* (2). The peak of the total number of particles that corresponds with inhalation in each breathing cycle was detected. The height of each peak was defined as the difference of total particle count between the peak and the previous minimum (inhalation phase). Only samples laying within the top 10% of a peak were included in the analysis assuming this represents the actual inhaled air that is not yet filtered by the lung. The background concentration time series were corrected with a moving average spanning over 10 samples (i.e. 10 s) to account for natural fluctuations. The ratio of the particle concentration inside the mask c_{in} and in the background c_{bg} was calculated for all particle size ranges for each of the included samples. Using only samples from inhalation corrects the total inward leakage by an average of +8%. For each subject, the mean *TIL* was determined over all inhalation cycles. The median of the total inward leakage values of the subjects was calculated for each mask case (i) to (v). Lastly, the particle size - total inward leakage median curves were smoothed with a moving average spanning over three data points.

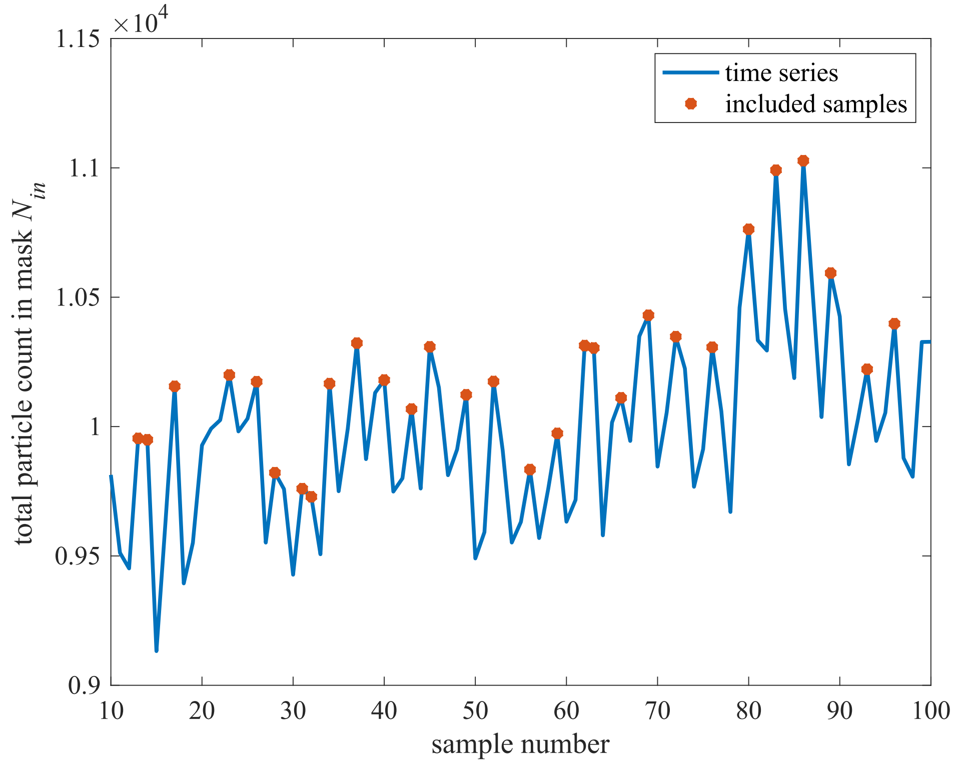

Fig. S4. Example of measured total particle concentration as a function of sample number. Highlighted (red dots) are those samples which correspond to an inhalation period as defined in Subsection E.2. Only samples with sample number >10 were included to account for possible remaining particles in the tubes and diffusion dryers.

2. Results

A. Filter penetration. Figure S5 shows the measured filter penetration of three different cloth, eight surgical and five FFP2 masks. Between all the masks examined in this study, the cloth masks have the highest filter penetration with the maximum penetration for particles around 0.3 µm diameter, whereas the FFP2 masks have the lowest penetration as shown in Fig. S5. On average the cloth mask filters have a penetration of 85% for particles with a diameter of 300 nm with the worst-performing mask filter material reaching a penetration close to 90%. These results are within the range of penetration values measured previously(3–6). All the examined FFP2 masks are below the 6% limit set by the EN 149:2001+A1:2009 standard (1). On average the filter penetration further decreases with particle size for particles larger than 50 nm, this tendency is much stronger for three of the tested FFP2 masks.

Surgical masks fall into two categories, four exhibit <12% filter penetration with maximum penetration for very small particles ($30 - 120$ nm) while the other four have filter penetration values almost as high as cloth masks. The latter category also shows the same *characteristic maximum filter penetration* at 0.3 µm as the cloth masks, ranging from 50% to 75%. The strongly varying results from previous studies (3, 7–9) agree with our finding that surgical masks exhibit vastly different filtering characteristics depending on the model.

The dotted lines in Fig. S5 show the filter penetration of particles through the filter material of the FFP2 and surgical masks that were used in the leakage experiments measured with penetration setup 2.The filter of the surgical mask has a

maximum filter penetration of 10% for particles of around 30 nm diameter. A smaller peak in the penetration of about 5% can be observed for particles with 0.3 µm diameter. For larger particles the filter penetration further decreases. The filter penetration of the FFP2 mask was smaller than 1.3% for all particle sizes and below 0.03% for particles larger than 30 nm. For increasing particle size the filter penetration decreases drastically for both types of masks. A more detailed discussion of the filter penetration results in comparison to existing literature can be found in the following section 2.A.1. It has to be noted that the flow velocities through the masks (and the equivalent breathing rates) vary by a factor of 4 between the two setups used in our penetration measurements. The flow rates in setup 1 are smaller than the one required by the European standard EN 149:2001+A1:200 (95 L min^{-1}) (1). Despite filter penetration being reported to depend on breathing flow rates and therefore flow velocity through the material (e.g. 7, 10–12) our results do not seem to be noticeably influenced by it (dotted lines overlap with dashed lines, Fig S5). The differences between different masks of a certain type exceed the potential influence of flow rate.

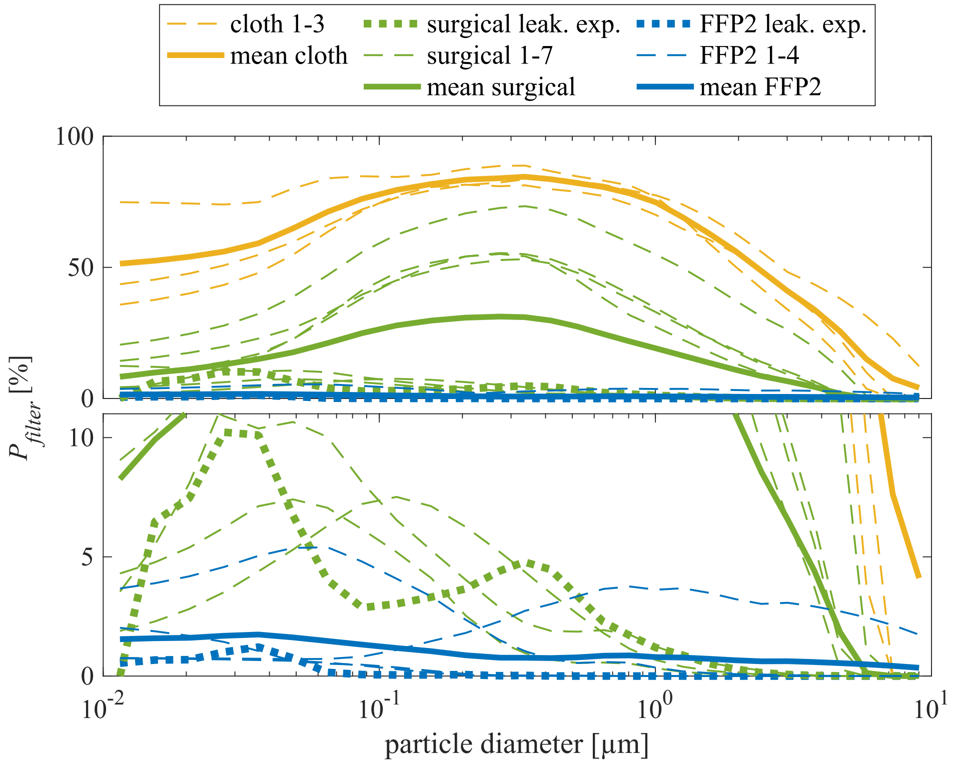

Fig. S5. Particle penetration as a function of particle size through the filter material of the tested cloth, surgical and FFP2 masks. Dashed lines show the results from measurements of filter penetration of different masks in setup 1. Dotted lines show the results from filter penetration measurements in setup 2 for the two masks that were used in the leakage experiment. The solid lines are the mean of each mask type.

A.1. Filter penetration literature comparison. The three measured cloth masks show the highest filter penetrations for all particle sizes. Their maximum penetration through the filter can be observed for particles of a size of around 300 nm. On average the cloth mask filters have a penetration of 85% for particles of that size with the worst-performing mask filter material reaching a penetration close to 90%. For particles >300 nm the filter penetration decreases with particle size so that all cloth masks have a filter penetration lower than 50% (on average 40%) for 3 µm and below 15% (on average just 5%) for the largest measured particles. Zangmeister *et al.* (3) measured the filtration efficiency of different cloths (and varying layer numbers) were measured. The highest filter penetration in the range of around 200 to 250 nm, which agrees with our findings. The maximum filter penetration for different cloth masks varied between 65 and close to 100% (cotton, polyester and blends of both were tested). Morais *et al.* (4) found the average maximal filter penetration for 300 nm sized particles for 54 examined cotton mask to be 68%. In a study by Konda *et al.* (5), even more, materials used for cloth masks are examined. The maximum filter penetration ranged from less than 10% for certain material combinations and up to over 90% for thinly woven (low thread per area ratio) cotton.

The filter penetration of the eight examined surgical masks varied much more than that of the examined cloth masks. Four of the masks (1-4) showed the characteristic maximum filter penetration at 300 nm that was also observed for the cloth masks. The peak filter penetration ranged from 50% to 75% for the four masks that showed a significant peak. Particles larger than 300 nm get filtered much better, the filter penetration drops to under 30% for all masks for particles with a diameter >3 µm. For the other four surgical (5-7, leakage exp. mask) masks, a filter penetration of less than 12% was observed throughout all particle sizes with peaks between 30 and 120 nm. The surgical mask examined by Zangmeister *et al.*. (3) showed a maximum of 70% filter penetration at a particle size of around 200 nm with increasing filter penetration with particle size before that peak and decreasing with particle size after that peak. This observation is very similar to what was found here for the surgical

mask 1-4. Grinshpun *et al.* (8) found a decreasing filter penetration for a surgical mask with particle size in the range from 0.04 to about 1 µm that was below 10% throughout which is comparable to our findings for surgical masks 5-7 and the surgical mask used in the leakage experiment. In a study by Balazy *et al.* (7), two surgical masks were compared regarding their filter penetration at two different flow rates (30 and 85 $\mathrm{L\,min^{-1}}$) for nanoparticles up to 80 nm. For both, the filter penetration increased with particle size for these small particles. However, one mask performed by a factor of approximately 4 better compared to the other one (at a higher flow rate a maximum of 85% compared to maximum of 21%). The filter penetration of different types of surgical masks was measured by Oberg *et al.* (9). They were categorized as "dental" or "hospital" masks and tested with spherical latex particles of three different sizes (0.9, 2 and 3.1 µm). For the dental masks, the filter penetration was as bad as up to 80% for the smallest spheres. The six tested hospital masks had however a filter penetration of less than 2% for all sizes. In all cases, the filter penetration decreased with particle size. These previous studies agree with our finding that surgical masks exhibit vastly different filtering characteristics depending on model. Generally, there seem to exists two different classes of filter materials for surgical masks within which the filter penetration is comparable over different studies.
The standard for FFP2 masks is that filter penetration should be less than 6% (at 95 $\mathrm{L\,min^{-1}}$, EN149:2001+A1:2009 (1)). All masks we examined lie within this limit. On average the filter penetration further decreases with particle size for particles larger than 50 nm, this tendency is much stronger for two (three with leakage exp. mask) out of the tested masks.In (13) and (14), the peak filter penetration for FFP2 masks is for particles between 30 50 nm. Most masks lie within the 6% leakage limit for all particle sizes, however for one FFP2 mask in (14) a maximum filter penetration of 17% was found in the peak particle size range. Further studies (7, 8, 12, 13) examined other comparable (N95, FFP3 and P100) respirators. The peak filter penetration was always found in the range of about 40 to 50 nm. Most masks were found to have a filter penetration that was within the defined limit of the respective respirator type, only a few showed higher filter penetrations in the peak particle range at high breathing flow rates.

B. Test experiment 1: Reading, mask inlet position. To be aware of potential sampling biases and factors that influence the total inward leakage a variety of test experiments were performed. These test experiments were not performed with the final experimental setup as it was continuously improved. Here, no dryers were used, a higher sampling flow rate of 6 $\mathrm{L\,min^{-1}}$ was used and the sensitivity correction did not account for particle losses in the tubes but only for the sensitivity of the OPS spectrometers themselves. Moreover, they were performed on very few subjects. Therefore, the absolute values given in the following sections have to be treated cautiously but general trends can be observed. Since subject number varied between 2 and 3, instead of the median over subjects, the mean values are given in the following. All investigated mask cases are shown for each potential factor but not all cases (i) to (v) were always investigated.

Figure S6 shows a comparison between nose breathing and reading for mask cases (i) to (iv). It can be seen that nose breathing leads to a higher measured total inward leakage in all cases. The observed differences range from an average factor of 2 for the adjusted case (i) to an average factor of over 9 for the taped mask case (iv). The detection of inhalation periods might not work as accurate in the reading case as inhalation is likely shorter and less deep. However, the significant differences shown in Fig. S6 can still be observed, when all samples (independent of breathing cycle) from the measurement time series are included in the analysis. It should also be mentioned that an increase in penetration with increasing particle diameter (as visible in the dashed blue curve of S6) is most likely the result of poor counting statistics in the large particle sizes.

As it is unclear how well the air inside a mask mixes, the inlet position of the sampling tube could affect measured in-mask concentration. To investigate this we compare two different vertical inlet positions in Fig. S7. The upper inlet (between the nose and upper lip) leads to higher mean total inward leakage except for the taped mask case (iv). However, the trend is not consistent for both subjects and the shown differences lie within the variability between mask fit of one subject (section 2D) and can therefore be deemed insignificant. Variability in measured total inward leakage of mask case (i) and (ii) between subjects is reduced when the upper inlet is used. For one subject the difference in case (iv) between the lower and upper inlet was very high (below 1% vs. over 20%) which can only be explained by a huge additional gap in one case and not by a sampling bias. The gap was probably caused by the helmet arm not being well adjusted in the test experiments. In following experiments, the helmet arm position was checked before every measurement both visually and by asking the subject whether they could feel any pulling or pushing from the inlet to avoid additional gaps.

C. Test experiment 2 : diffusion dryers, mouth breathing. Figure S8 shows the results for mask cases (i) to (iv) for a test experiment with 6 subjects compared to the results of the final experiment. In the test experiment no diffusion dryers were used and the sensitivity correction did not correct for particle losses in tubes. Adding the diffusion dryers lead to lower measured total inward leakages compared to the experiment without the dryer especially for larger particles with diameters of a few µm. The improved correction for particle losses (described in 1E) corrected the new measurements upwards and therefore can not be the reason for this discrepancy. The high humidity inside the mask can lead to growth of the size of the particles inside the mask. This shifts the whole *TIL* curve towards larger particles (to the right in Fig. S8). This hypothesis is also supported by the plateau in the results of the experiment without the dryer which can be observed for particles <700 nm whereas the *TIL* decreases monotonically throughout all measured particle ranges and the plateau is expected only for particles <300 nm (cf. Fig. S13). The surgical mask used in the two experiments was different. The filter penetration of the mask used in the experiment without dryers was smaller by at least a factor of 2 for all particle diameters >300 nm which can explain the deviation from the trend that not using a dryer leads to an overestimation of *TIL*. Moreover, in the test experiment overall an improvement due to the surgical mask worn on top of the adjusted FFP2 mask compared to the adjusted mask itself was

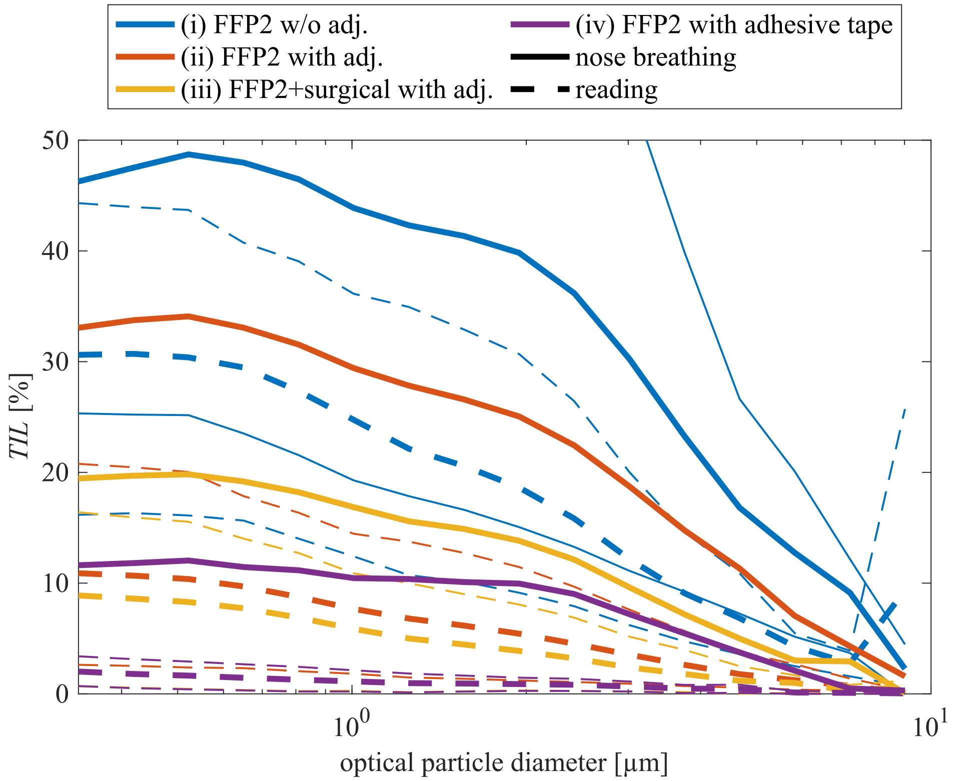

Fig. S6. Comparison between reading and nose breathing. Measurements were performed on three different subjects, the mean is shown. The sampling rate here was with $6\,\mathrm{L\,min^{-1}}$ higher than in the final experiment and the lower inlet was used.

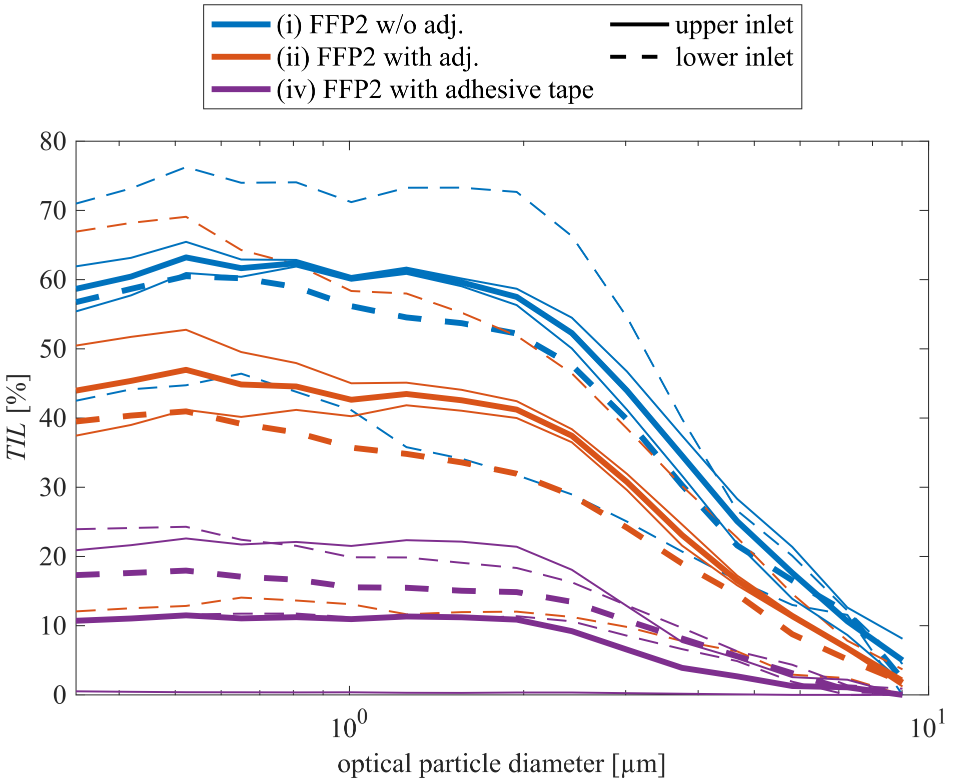

Fig. S7. Comparison between two different inlet positions. "upper" position is the position that was used in the final experiments: right between the upper lip and nose. "lower" was a tested position near/under the lower lip. Measurements were performed on two different subjects, the mean is shown. The thin lines show the minimum and maximum TIL over different subjects. The sampling rate here was with $6\,\mathrm{L\,min^{-1}}$ higher than in the final experiment.

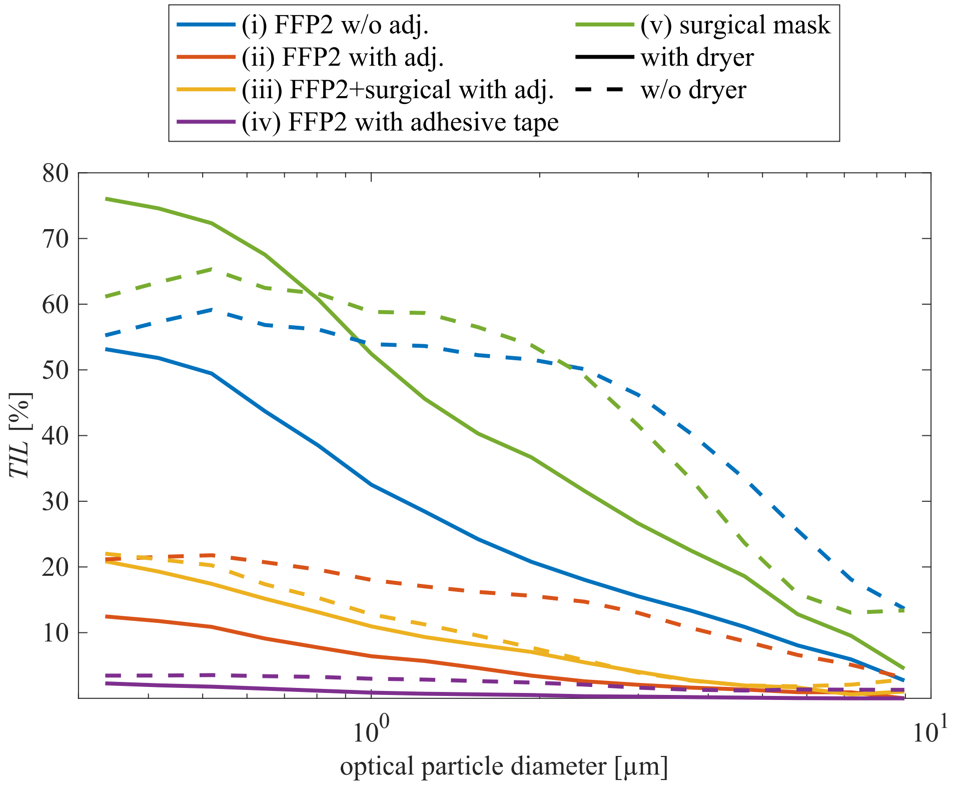

Fig. S8. Comparison of median leakage of final experiment (7 subjects, in-mask and background samples dried, improved sensitivity correction) to those of a test experiment (6 subjects, no dryers, correction does not take particle losses into account). In the final experiment an additional mask worn on top of the FFP2 facepiece overall increased the leakage, whereas the opposite was observed in test-experiment. Here, no errorbars are shown for better visibility. The uncertainties of TIL in the final experiment are shown in the main paper (Fig.2).

observable whereas the opposite was found in the final experiment. This leads to the hypothesis that an additional surgical mask can also sometimes close face seal leaks that were present when the mask was worn alone by pressing it tighter onto the face. The results for wearing a surgical mask on top of an FFP2 mask are therefore inconclusive.

In the experimental setup without diffusion dryers, the influence of mouth breathing compared to nose breathing was investigated for two out of the seven subjects. For the not adjusted and adjusted FFP2 mask breathing through the mouth leads to higher total inward leakage on average, whereas in the taped mask case (iv) it leads to lower total inward leakage. In Figure S9 the mean of the two subjects is shown. These trends can be observed for both subjects individually as well. Since the mask was not taken off between nose and mouth breathing measurements these differences can not be associated with a different fit of the mask and therefore only result from the different kind of breathing.

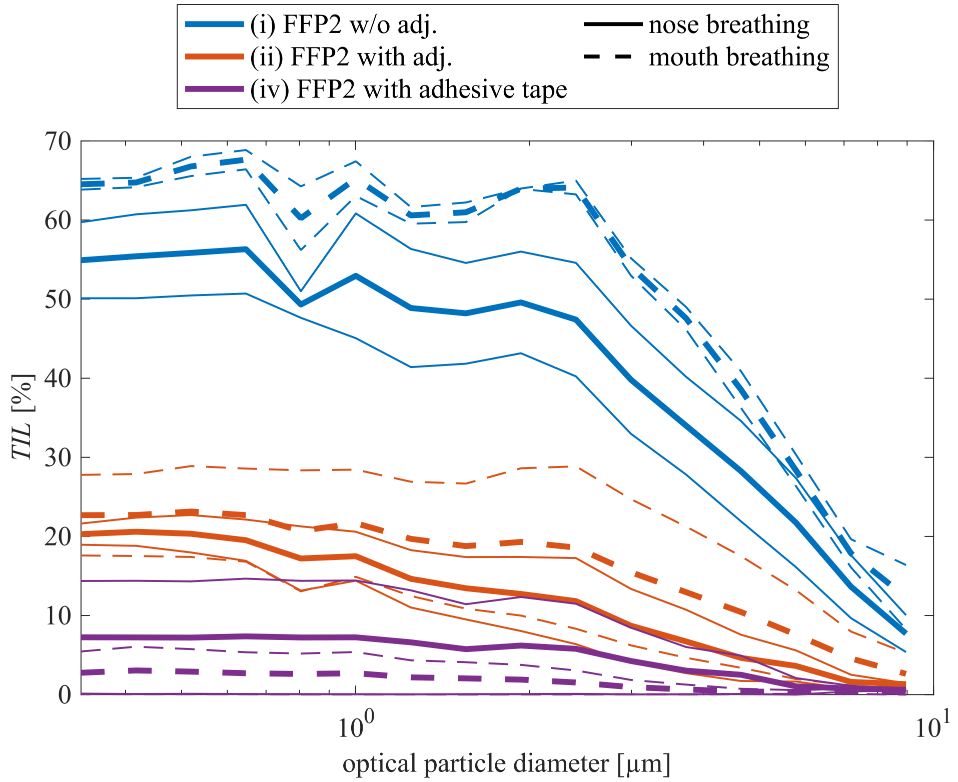

Fig. S9. Comparison between mouth nose breathing. Measurements were performed on three different subjects, the mean is shown. The thin lines show the minimum and maximum TIL over different subjects. The setup is the one that was used in the final experiment but without diffusion dryers.

D. Inward leakage variability for the same subject. The cases (i), (ii) and (iv) were tested two separate times for two out of the seven subjects (Fig. S10), the mask was taken off in between measurements. We found, that the total inward leakage of each mask case and therefore the fit varies from time to time even if the mask is donned by the same subject. As mentioned in the main paper, the results vary by 40% for the FFP2 mask without adjustment and up to factor 16 (factor 16 for one subject that also stated an unusually high fit of the taped mask (always below 7%), and factor 2 for the other subject (always below 2%)) for the FFP2 mask with adhesive tape on the nosepiece from time to time for the same mask/subject combination. The relative variability increases with better fitting mask cases.

E. Influence of facial hair. In the final experiment, the tape on the nosepiece was the most leak-tight case for all subjects except one, for whom it performed as good as the adjusted mask and the adjusted mask worn with an additional surgical mask on top. A possible explanation could be the beard of the subject which leads to more leakage around the cheek and the chin area which do not get closed by the tape. To further investigate the influence of facial hair the mean leakage for the three FFP2 mask cases (i), (ii) and (iv) are shown in Fig. S11 for two different groups: subjects with facial hair and subjects without facial hair. Since subject number in each group was only 3 and 4 the mean as opposed to the median was chosen here. It can be seen that facial hair increases the total inward leakage and therefore the face seal leakage in all cases. Having no facial hair leads to an average improvement of over 30% with a non-adjusted mask, an improvement by over 80% with an adjusted mask and by more than a factor of 8 with a mask taped to the nosepiece. Overall, these exact values have to be treated carefully as the total number of subjects for each category (with or w/o facial hair) was very low and as discussed variability in total inward leakage was generally very high. A general trend can ,however, be observed from our results. For the total inward leakage of the surgical mask, facial hair made the least difference (around 16% improvement with no facial hair). This can be explained by the fact that surgical masks are not designed to fit tight and seal to the face.

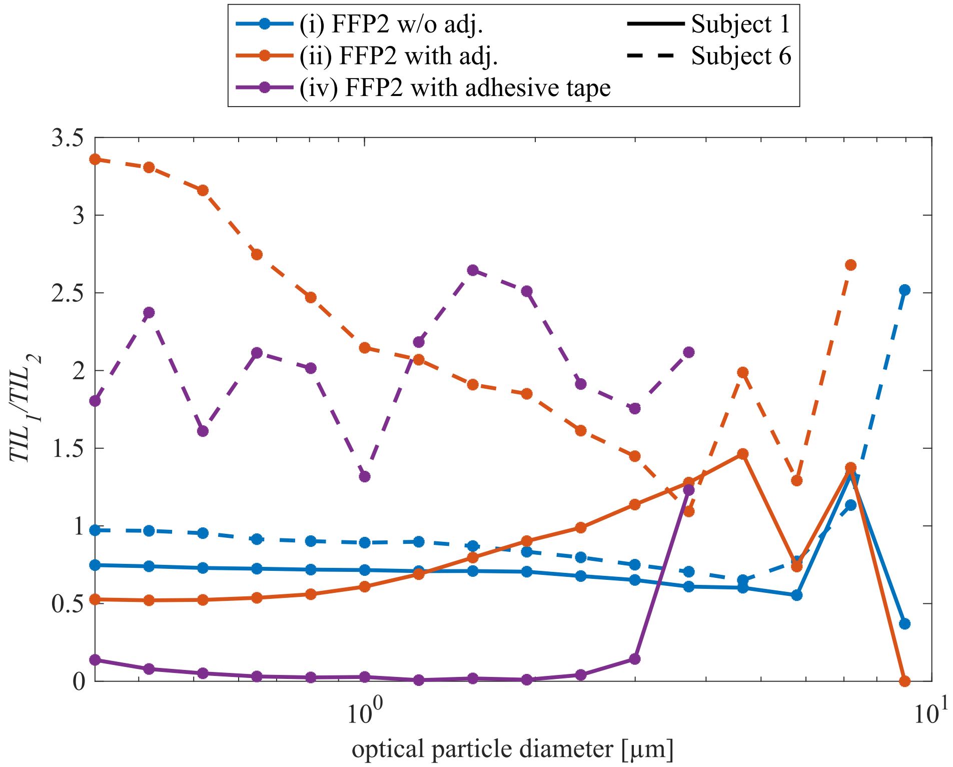

Fig. S10. Two separate measurements, in between which the mask was taken off, were performed with the final setup (Fig. S3) for the three FFP2 mask cases (i) not adjusted, (ii) adjusted and (iv) with adhesive tape for the two subjects S1 and S6. The graph shows the first measurement normalized by the second to show variability in the total inward leakage for a single subject.

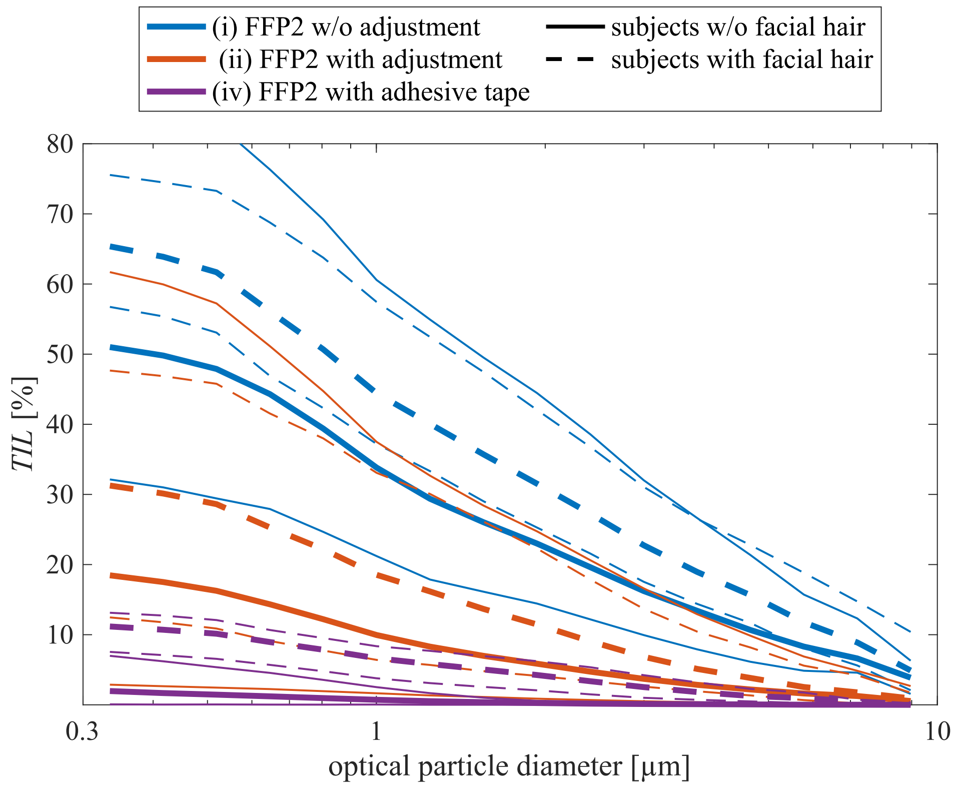

Fig. S11. Comparison of FFP2 mask-wearing case for subjects with facial hair and without measured in the final setup (Fig. S3). Facial hair increases the mean total inward leakage of the FFP2 mask in all cases. The thin lines show the minimum and maximum TIL over different subjects.

F. Literature comparison of total inward leakage. In the following, a detailed comparison with existing literature of total inward leakage of masks is given. Let us first recapitulate the prior knowledge in a little more detail. Although the literature on mask efficacy is quite extensive, we felt it was necessary to perform our own measurements specifically on TIL for several reasons: size-dependent TIL data are needed to calculate the risk of infection. As a result, a number of studies in which size-dependent results are not presented could not be used (e.g. 9, 15–21). In order to correctly assess real-life situations, tests on manikins (e.g. 10, 22) must be compared with those on volunteers. Tests in which TIL is simulated by artificially introduced leakage (e.g. 23–31) are scientifically interesting, but cannot be applied concretely because the range of natural leakages must be known. The data from (8, 32, 33), in which TIL tests on human subjects with non-artificial leaks were conducted, are each limited to particle diameters <1.3 µm, but data for larger particles are needed for infection risk assessment. The TIL data from N95 respirators from (34) cover particle sizes up to 10 µm, but not the size range <0.7 µm where the highest TIL is expected. In some of the studies with human subjects, the subjects best fitted their masks before the measurements using test equipment (e.g. 34, 35). However, the common user does not have access to such equipment before using a face mask, nor is the user professionally trained in how best to wear a mask. Therefore, we use exemplar TIL measurements of subject and mask variability. To represent the particle size range relevant to infection risk, we chose the size range from 300 nm , where we expect maximum TIL (see SI, fig.12 and fig.13), to 10 µm. As it is shown in Fig. 2, for large particles, the TIL is negligible with conventional mask design. Our TIL measurement setup is quite similar to the one used in the EN 13274-1:2001 (36) standard. In our experiments we use dolomite dust as test particles to faithfully cover the particle size range from 300 nm to 10 µm.

Following these arguments, the direct comparison here is only made with studies showing size-dependent TIL results for naturally occurring face seal leaks on surgical and FFP2/N95 face masks. Figure S12 shows our results regarding the leakage of a surgical mask compared to findings from previous studies and Fig. S13 our results for the FFP2 mask compared to existing literature on leakage of FFP2 but also the comparable N95 masks. For the direct comparison in the figure, we further limit the shown literature to selected studies that were performed on human subjects but results from manikin studies with natural leakage are also discussed in the following. In a study by He *et al.* (10) a surgical mask was donned on a manikin without

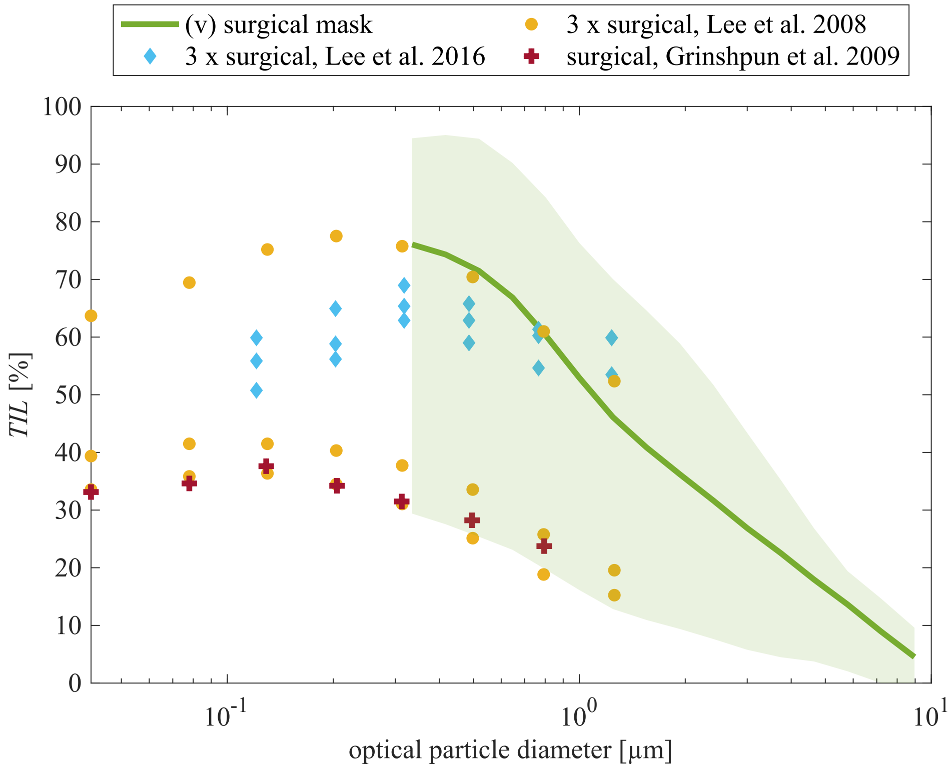

Fig. S12. Our measured median total inward leakage of an adjusted surgical mask (with minimum and maximum represented by shaded area) compared to previous studies on total inward leakage of surgical masks on human subjects: Lee *et al.* 2008 (33), Lee *et al.* 2016 (32) and Grinshpun *et al.* 2009 (8).

any artificial leaks. Total inward leakage was up to around 30% of particles around 0.1 µm for the surgical mask at a MIF of 15 L min^{-1}. Similar results were found in human subject studies by Grinshpun *et al.* (8) where the TIL maximum of a surgical mask was up to nearly 40% and by Lee *et al.* (33) where the protection factor of three surgical masks was measured to be average at 2.4 which corresponds to TIL of 42%. One of the surgical masks performed worse than the other two, for this mask the findings from Lee *et al.* agree well with our results for the surgical mask. In a study by Lee *et al.* (32) the maximum leakage was found to be between 60 and 70%. In all studies, the maximal leakage was found to be between approximately 0.1 and 0.3 µm. Compared to the findings by Lee *et al.* (32) for well adjusted cone-shaped FFP2 face masks worn by humans, the observed leakage for the adjusted case here (maximum of 12.5% for particles with an average diameter of 0.34 µm, reduces

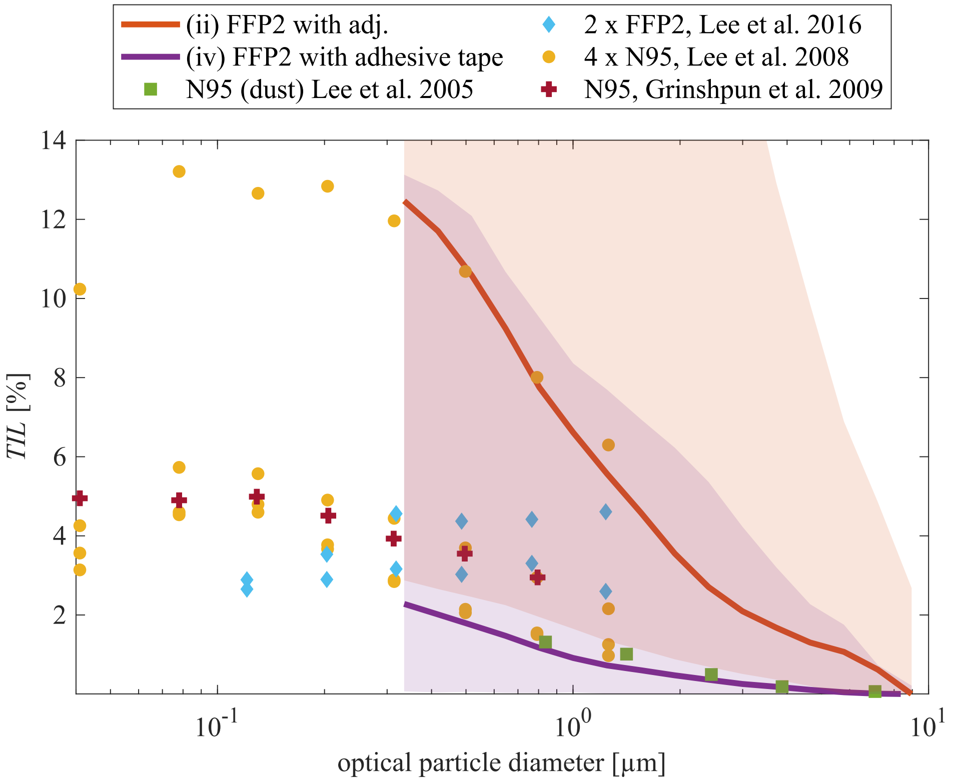

Fig. S13. Our measured median total inward leakage of an adjusted (red) and taped (purple) FFP2 mask (with minimum and maximum represented by shaded areas) compared to a previous study on total inward leakage of FFP2 masks on human subjects Lee *et al.* 2016 (32) and of N95 masks by Lee *et al.* 2005 (34), Lee *et al.* 2008 (33) and Grinshpun *et al.* 2009 (8).

to 10% if subjects with facial hair are excluded) is larger (cf. Fig. S13). Lee *et al.* observed median protection factors of over 20 (two different FFP2 masks tested on 30 subjects) which means leakage of less than 5% without a strong particle size dependency in the measured range. Other studies from Lee et. al (33, 34) and ref. (8) where N95 masks (comparable to FFP2) were tested show total inward leakages in the same order of magnitude (Fig. S13). He *et al.* (10) found leakages of an N95 mask less than 3% for an N95 with different breathing rates and frequencies on a manikin. However, our results for the adjusted FFP2 mask seem to agree very well with the worst performing N95 respirator a study by Lee *et al.* (33). When the FFP2 mask was taped to the nosebridge, the leakage is less than what was observed in all studies referenced in Fig. S13. It has to be noted that, the leakage in the referenced studies (8, 32, 33) was measured while the subjects performed different fit testing exercises (head movements, different breathing patterns) and in ref. (34) while subjects were working on agricultural farms (plus they had to pass a quantitative fit test before with FF> 100), it is unclear whether the taped FFP2 mask would still perform better under similar conditions. Our subjects underwent no quantitative or qualitative fit testing procedures before the total inward leakage measurements. There was a set protocol for how the subjects should don and adjust the mask including checking whether the subject could feel any leaks. Besides this, no subject was properly trained on how to fit a mask. Hill *et al.* (22) found even higher total inward leakage values for a N95 and KN95 respirator donned on a manikin of over 80% for particle diameters of 125 nm measured at $12\,\mathrm{L\,min^{-1}}$. It can be assumed that the mask was not adjusted to the manikin. However, those values are even higher than what we found for the not adjusted mask case.

G. Combined/effective penetration surgical mask. Figure S14 shows the combined penetration for the *mask-SS* case. The influence of shrinkage ($w = 4$ compared to $w = 1$), the leakage of the mask (with leakage, adj. compared to leak-free) and of considering respiratory tract deposition (effective combined penetration) is similar to what we observed and discussed for the *mask-FF* case in the main paper (section B). However, the combined penetrations of the *mask-SS* case have different magnitudes and are much higher for all cases. This is caused by a higher filter penetration and higher total inward leakage of the surgical mask.

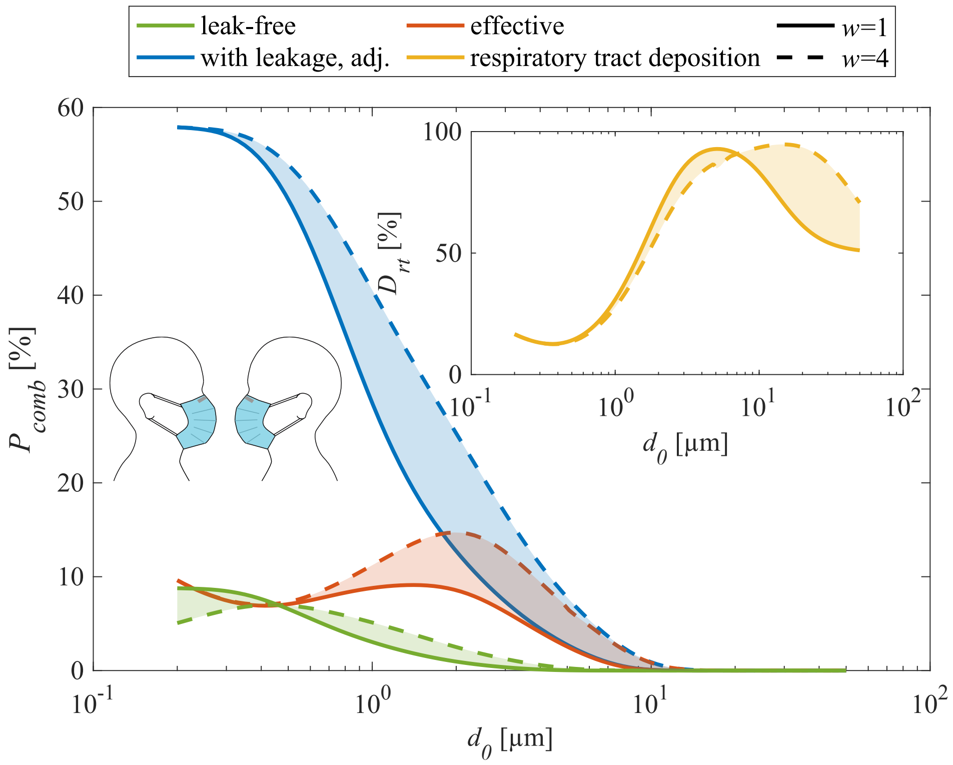

Fig. S14. Combined penetration values when both infectious and susceptible are wearing surgical masks (combined penetration for mask-FF scenario is shown in main paper), and at different shrinkage factors of $w = 1$, i.e. no shrinkage, and $w = 4$ as a function of particle diameter at exhalation, i.e. wet diameter. *Leak-free* curves correspond to $P_{\text{comb,leak-free}} = P_{in}P_{ex}$, *With leakage, adj* curves correspond to $P_{\text{comb, adj}} = [P_{in} + L_{in}]\,[P_{ex} + L_{ex}]$ and *effective* curves correspond to $P_{\text{comb, effective}} = D_{rt}\,[P_{in} + L_{in}]\,[P_{ex} + L_{ex}]$. Respiratory tract deposition D_{rt} is shown in the inset for $w = 1$ and $w = 4$.

H. Combined/effective mask penetration for selected asymmetric cases. Fig. S15 shows penetration values for the "FS" (infectious: adjusted FFP2, susceptible: surgical) and "SF" (infectious: surgical, susceptible: adjusted FFP2) cases shown in Fig. 6. It can be seen that the effective penetration is slightly lower when the better mask is worn by the infectious subjects (FS case) than when the better mask is worn by the susceptible subjects (SF case). The maximum difference is about 0.75% and occurs at d_0 =2.2 µm. However, as explained in the main text, this minor difference should not be over-interpreted, as

the uncertainties in the assumptions underlying the derivation (especially TIL = TOL) might be higher than the observed differences.

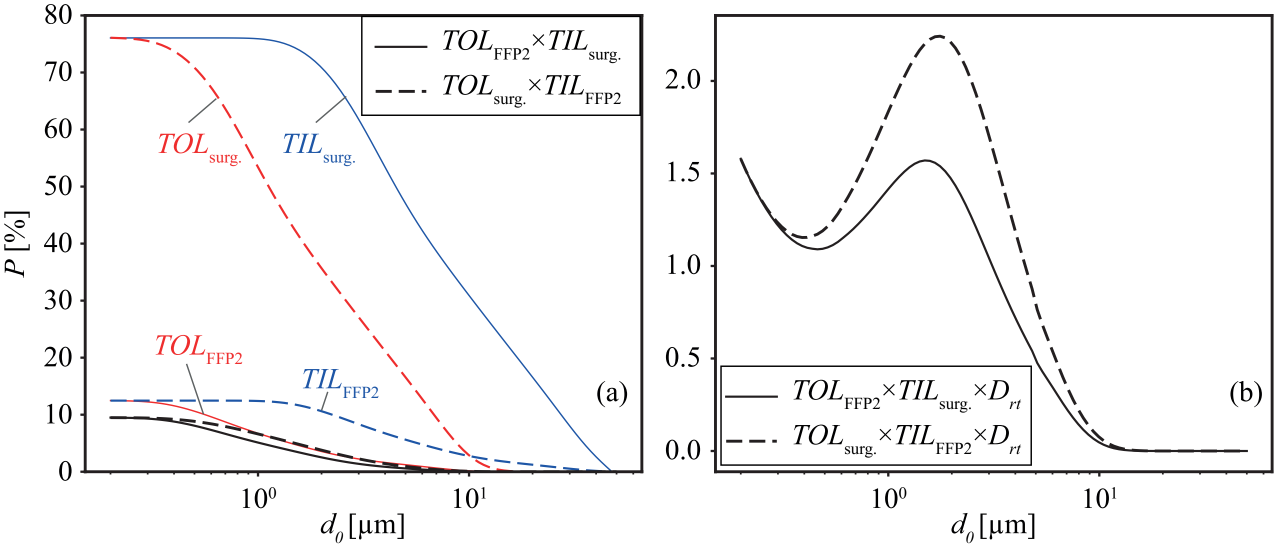

Fig. S15. (a) combined and (b) effective penetrations for selected mask scenarios in which infectious and susceptible wear different kinds of masks while $w = 4$. $TOL_{\mathrm{FFP2}} \times TIL_{\mathrm{surg.}}$ corresponds to "FS" in Fig. 6 (infectious wearing an adjusted FFP2 and susceptible a surgical mask), $TOL_{\mathrm{surg.}} \times TIL_{\mathrm{FFP2}}$ corresponds to "SF" in Fig. 6 (infectious wearing a surgical and susceptible an adjusted FFP2 mask) and D_{rt} is the intake/deposition efficiency of the susceptible respiratory tract.

I. Leakage results for individual subjects. In Fig. 2 of the main paper we show the median total inward leakage over all 7 subjects. Here, we show the results for each subject individually. As discussed in the main paper there is a large scatter in mask fit depending on the sujects. However, it can be seen that the general trends observed for the median also apply to the individual results.

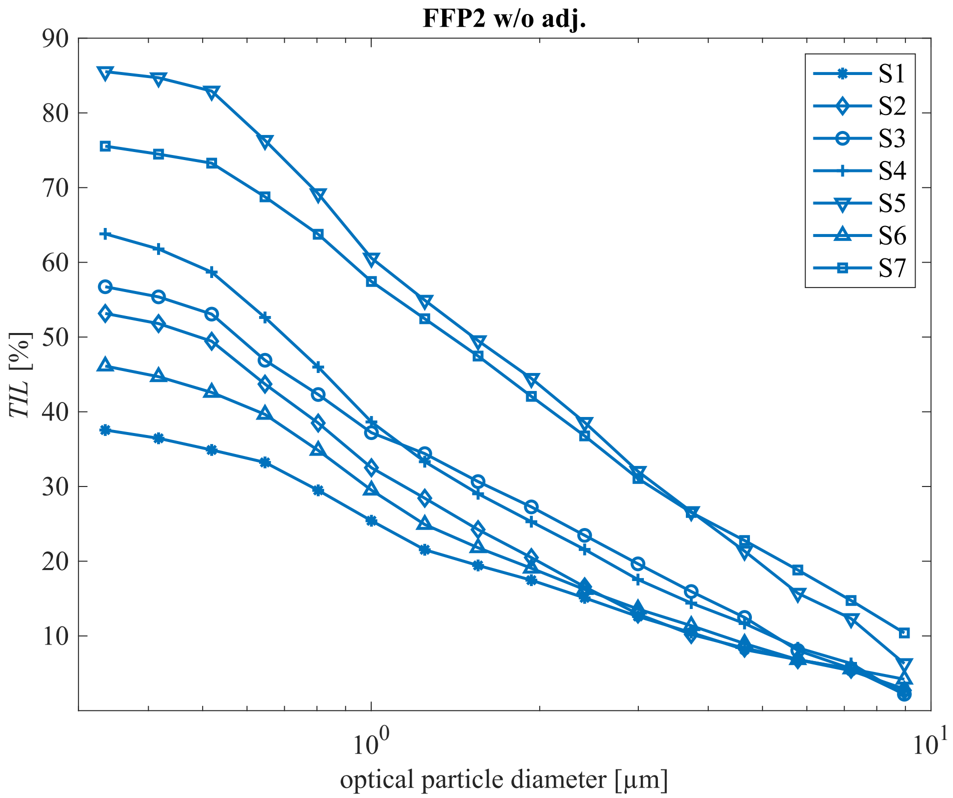

Fig. S16. Mean TIL for subjects $S1$ to $S7$ wearing a FFP2 mask without adjustment as a function of particle size.

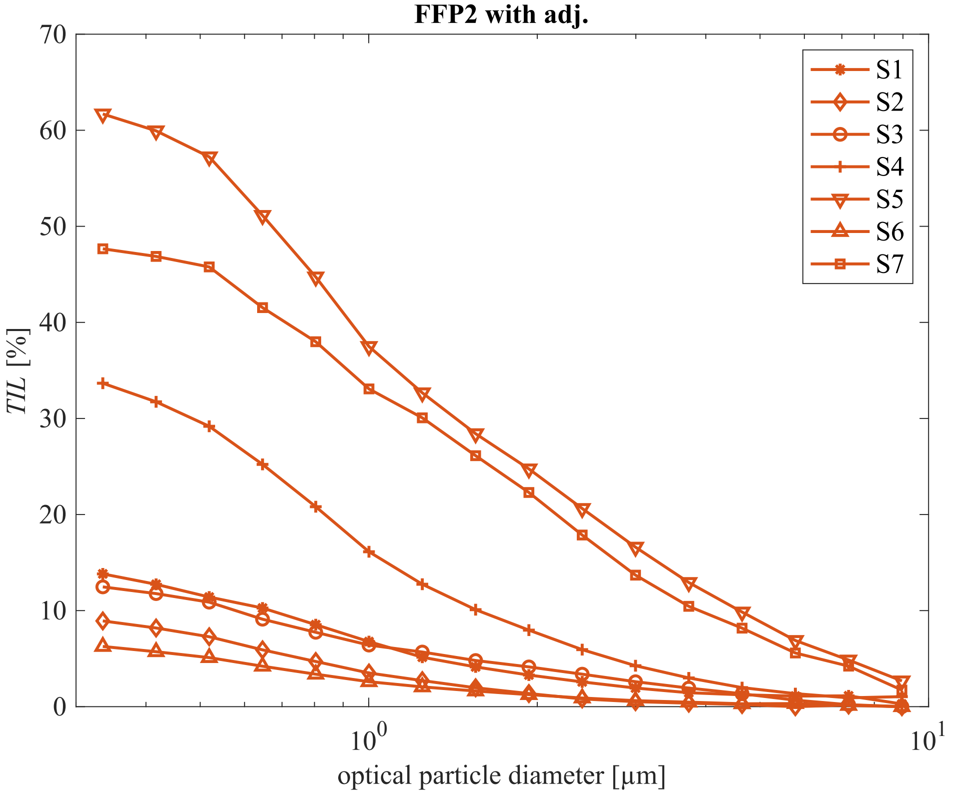

Fig. S17. Mean TIL for subjects $S1$ to $S7$ wearing a FFP2 mask with adjustment as a function of particle size.

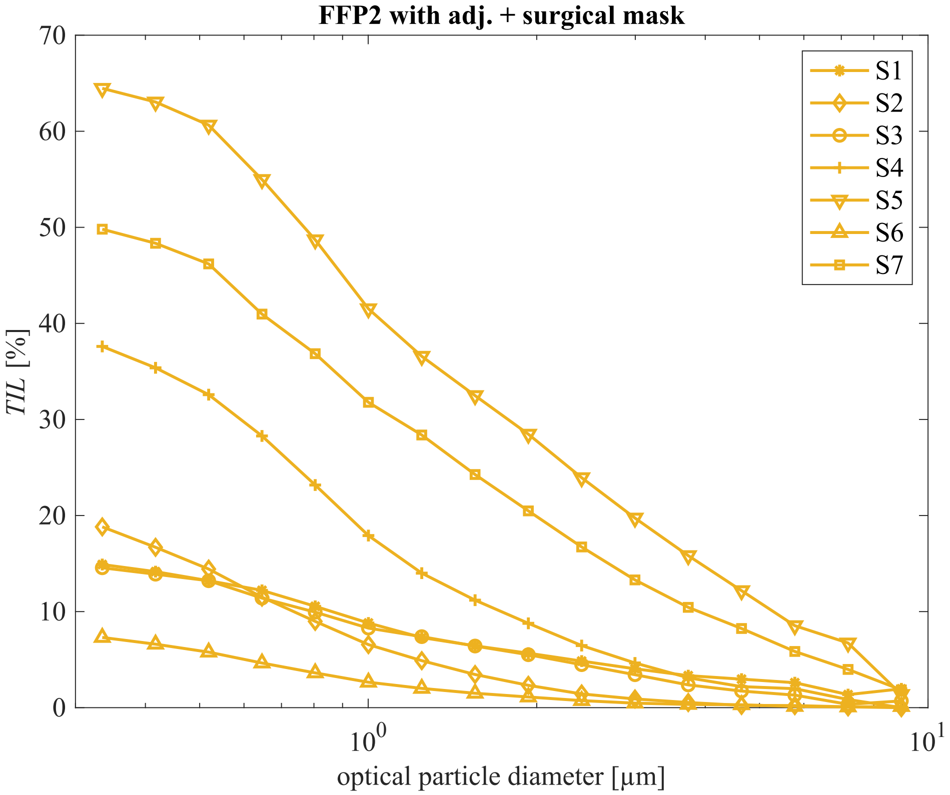

Fig. S18. Mean TIL for subjects $S1$ to $S7$ wearing a FFP2 mask with adjustment with an adjusted surgical mask on top as a function of particle size.

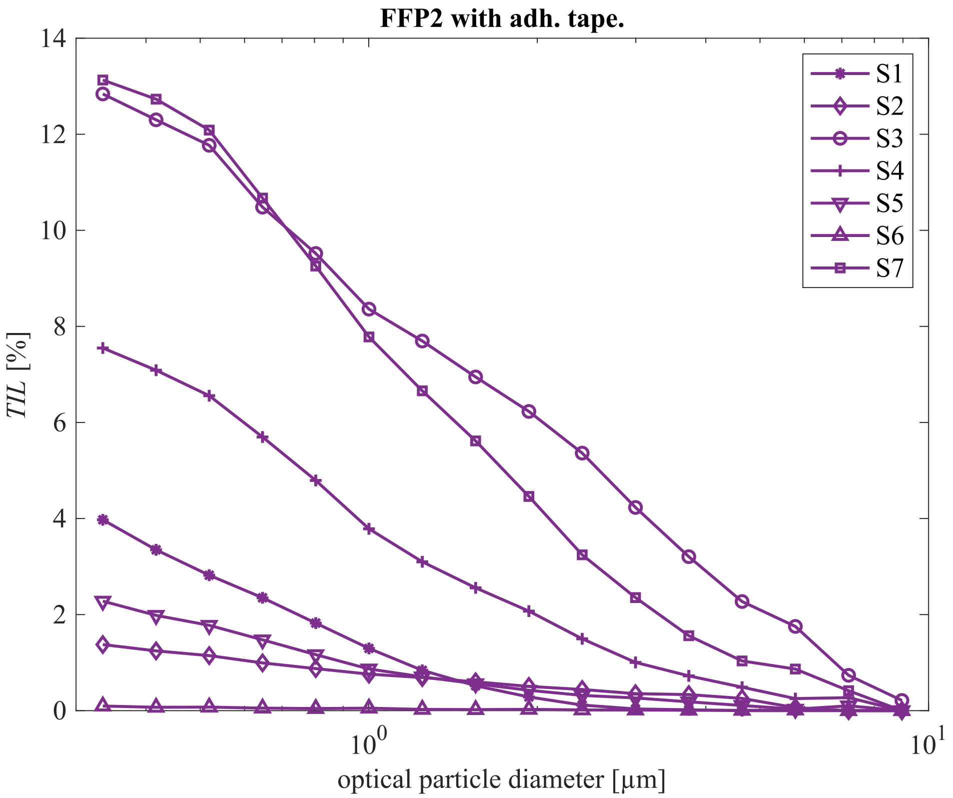

Fig. S19. Mean TIL for subjects $S1$ to $S7$ wearing a FFP2 mask with adhesive tape on the nose bridge as a function of particle size.

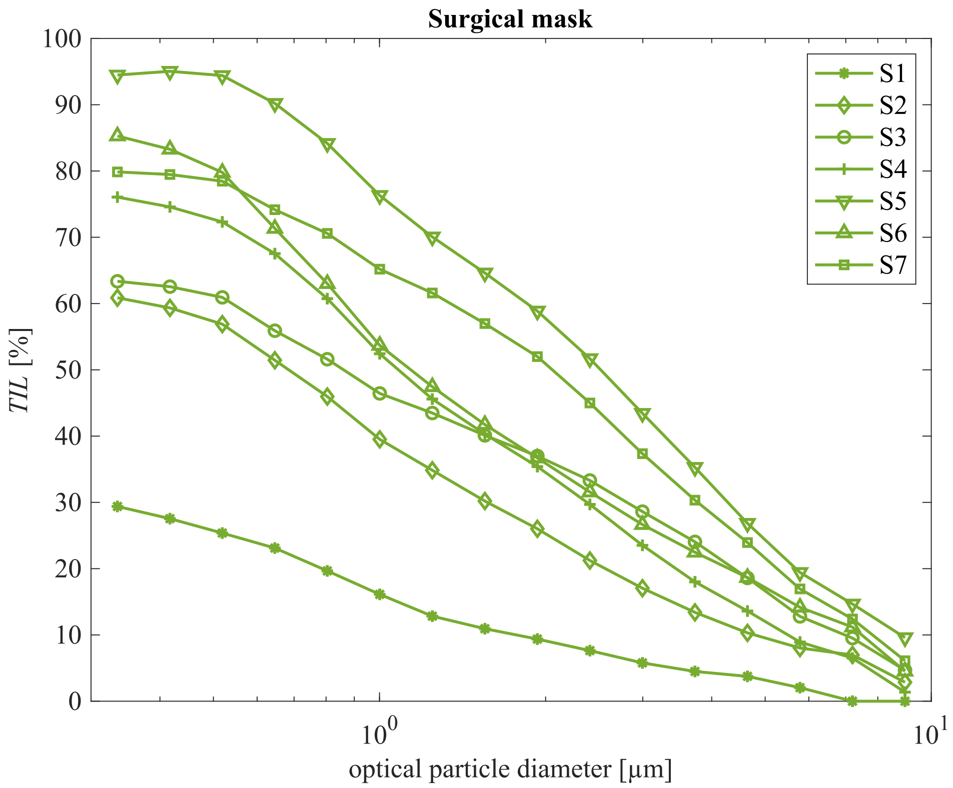

Fig. S20. Mean TIL for subjects $S1$ to $S7$ wearing a surgical mask with adjustment as a function of particle size.

References

1. European Committee for Standardization, Respiratory protective devices - Filtering half masks to protect against particles - Requirements, testing, marking (2009) EN 149:2001+A1.
2. WR Myers, J Allender, R Plummer, T Stobbe, Parameters that bias the measurement of airborne concentration within a respirator. *Am. Ind. Hyg. Assoc. J.* **47**, 106–114 (1986).
3. CD Zangmeister, JG Radney, EP Vicenzi, JL Weaver, Filtration efficiencies of nanoscale aerosol by cloth mask materials used to slow the spread of SARS-CoV-2. *ACS nano* **14**, 9188–9200 (2020).
4. FG Morais, et al., Filtration efficiency of a large set of COVID-19 face masks commonly used in Brazil. *Aerosol Sci. Technol.*, 1–15 (2021).
5. A Konda, et al., Aerosol filtration efficiency of common fabrics used in respiratory cloth masks. *ACS nano* **14**, 6339–6347 (2020).
6. F Drewnick, et al., Aerosol filtration efficiency of household materials for homemade face masks: Influence of material properties, particle size, particle electrical charge, face velocity, and leaks. *Aerosol Sci. Technol.* **55**, 63–79 (2021).
7. A Bałazy, et al., Do N95 respirators provide 95% protection level against airborne viruses, and how adequate are surgical masks? *Am. journal infection control* **34**, 51–57 (2006).
8. SA Grinshpun, et al., Performance of an N95 filtering facepiece particulate respirator and a surgical mask during human breathing: two pathways for particle penetration. *J. occupational environmental hygiene* **6**, 593–603 (2009).
9. T Oberg, LM Brosseau, Surgical mask filter and fit performance. *Am. journal infection control* **36**, 276–282 (2008).
10. X He, T Reponen, RT McKay, SA Grinshpun, Effect of particle size on the performance of an N95 filtering facepiece respirator and a surgical mask at various breathing conditions. *Aerosol Sci. Technol.* **47**, 1180–1187 (2013).
11. X He, T Reponen, R McKay, SA Grinshpun, How does breathing frequency affect the performance of an N95 filtering facepiece respirator and a surgical mask against surrogates of viral particles? *J. occupational environmental hygiene* **11**, 178–185 (2014).
12. A Bałazy, et al., Manikin-based performance evaluation of N95 filtering-facepiece respirators challenged with nanoparticles. *Annals Occup. Hyg.* **50**, 259–269 (2006).
13. S Rengasamy, BC Eimer, RE Shaffer, Comparison of nanoparticle filtration performance of NIOSH-approved and CE-marked particulate filtering facepiece respirators. *Annals Occup. Hyg.* **53**, 117–128 (2009).
14. N Serfozo, et al., Size-resolved penetration of filtering materials from CE-marked filtering facepiece respirators. *Aerosol Air Qual. Res.* **17**, 1305–1315 (2017).
15. LE Bowen, Does that face mask really protect you? *Appl. biosafety* **15**, 67–71 (2010).
16. U Pauli, S Karlen, K Summermatter, The importance of fit-testing particulate filtering facepiece respirators! *Appl. Biosaf.* **19**, 184–192 (2014).
17. RB Lawrence, MG Duling, CA Calvert, CC Coffey, Comparison of performance of three different types of respiratory protection devices. *J. occupational environmental hygiene* **3**, 465–474 (2006).
18. A Lai, C Poon, A Cheung, Effectiveness of facemasks to reduce exposure hazards for airborne infections among general populations. *J. Royal Soc. Interface* **9**, 938–948 (2012).
19. S Rengasamy, et al., Total inward leakage measurement of particulates for n95 filtering facepiece respirators—a comparison study. *Annals occupational hygiene* **58**, 206–216 (2014).
20. M Van der Sande, P Teunis, R Sabel, Professional and home-made face masks reduce exposure to respiratory infections among the general population. *PloS one* **3**, e2618 (2008).
21. J Gawn, M Clayton, C Makison, B Crook, Evaluating the protection afforded by surgical masks against influenza bioaerosols (Health and Safety Executive RR619) (2008).
22. WC Hill, MS Hull, RI MacCuspie, Testing of commercial masks and respirators and cotton mask insert materials using SARS-CoV-2 virion-sized particulates: comparison of ideal aerosol filtration efficiency versus fitted filtration efficiency. *Nano Lett.* **20**, 7642–7647 (2020).
23. WC Hinds, G Kraske, Performance of dust respirators with facial seal leaks: I. Experimental. *Am. Ind. Hyg. Assoc. J.* **48**, 836–841 (1987).
24. WR Myers, H Kim, N Kadrichu, Effect of Particle Size on Respirator Faceseal Leakage, (WEST VIRGINIA UNIV MORGANTOWN DEPT OF INDUSTRIAL ENGINEERING), Technical report (1990).
25. K Willeke, et al., Filtration, loading and faceseal leakage characteristics of filtering facepieces. *J. Aerosol Sci.* **23**, 749–752 (1992).
26. A Weber, et al., Aerosol penetration and leakae characteristics of masks used in the health care industry. *Am. journal infection control* **21**, 167–173 (1993).
27. C Chen, K Willeke, Characteristics of face seal leakage in filtering facepieces. *Am. Ind. Hyg. Assoc. J.* **53**, 533–539 (1992).
28. KJ Cho, et al., Large particle penetration through N95 respirator filters and facepiece leaks with cyclic flow. *Annals occupational hygiene* **54**, 68–77 (2010).
29. S Rengasamy, BC Eimer, Total inward leakage of nanoparticles through filtering facepiece respirators. *Annals occupational hygiene* **55**, 253–263 (2011).
30. S Rengasamy, BC Eimer, Nanoparticle penetration through filter media and leakage through face seal interface of N95 filtering facepiece respirators. *Annals occupational hygiene* **56**, 568–580 (2012).
31. S Rengasamy, BC Eimer, J Szalajda, A quantitative assessment of the total inward leakage of NaCl aerosol representing

submicron-size bioaerosol through N95 filtering facepiece respirators and surgical masks. *J. occupational environmental hygiene* **11**, 388–396 (2014).
32. SA Lee, et al., Particle size-selective assessment of protection of European standard FFP respirators and surgical masks against particles-tested with human subjects. *J. healthcare engineering* **2016** (2016).
33. SA Lee, SA Grinshpun, T Reponen, Respiratory performance offered by N95 respirators and surgical masks: human subject evaluation with NaCl aerosol representing bacterial and viral particle size range. *Annals Occup. Hyg.* **52**, 177–185 (2008).
34. SA Lee, et al., Respiratory protection provided by N95 filtering facepiece respirators against airborne dust and microorganisms in agricultural farms. *J. Occup. Environ. Hyg.* **2**, 577–585 (2005).
35. SA Lee, et al., Laboratory and field evaluation of a new personal sampling system for assessing the protection provided by the N95 filtering facepiece respirators against particles. *Annals Occup. Hyg.* **49**, 245–257 (2005).
36. European Committee for Standardization, Respiratory protective devices — Methods of test — Part 1: Determination of inward leakage and total inward leakage (2001) EN 13274-1:2001.
